# Calcium‐Mechanochemistry Enabled Ketone Synthesis From Organic Iodides and Carboxylic Acids

**DOI:** 10.1002/advs.76440

**Published:** 2026-07-09

**Authors:** Mengyao Pei, Zekun Yang, Xueyan Yang, Yufang Yang, Yangyang Shen, Jiemin Wang, Peile Ma, Zezhu Li, Xiaoliang Zheng, Xiaofeng Wei

**Affiliations:** ^1^ School of Pharmacy Xi'an Jiaotong University Xi'an China; ^2^ School of Chemical Engineering and Pharmacy Pharmaceutical Research Institute Wuhan Institute of Technology Wuhan China; ^3^ Xi'an Jiaotong University Suzhou Academy Suzhou China; ^4^ Department of Pharmacy The First Affiliated Hospital of Xi'an Jiaotong University Xi'an Shaanxi China; ^5^ Frontier Institute of Science and Technology Xi'an Jiaotong University Xi'an China; ^6^ Department of Chemistry University College London London UK; ^7^ Zhejiang Key Laboratory of Tumor Molecular Diagnosis and Individualized Medicine School of Basic Sciences and Forensic Medicine Hangzhou Medical College Hangzhou Zhejiang China

**Keywords:** ketone synthesis, mechanochemistry, organocalcium reagents

## Abstract

Organocalcium reagents possess a reactivity profile distinct from that of classical organolithium and Grignard reagents, yet their synthetic potential remains underexplored. Here we report that mechanochemically generated organocalcium species exhibit an unusual chemoselectivity toward carboxylate salts, enabling the direct and selective transformation of carboxylates into ketones without overaddition, a persistent limitation of conventional organometallic chemistry. Commercially available calcium metal is directly activated by ball milling with aryl iodides to generate reactive organocalcium intermediates under solvent‐minimized and ambient conditions. This protocol exhibits broad substrate scope, excellent chemoselectivity, and high functional‐group tolerance. Moreover, the reaction can be extended to the direct use of carboxylic acids, enabling a one‐step synthesis of pharmaceutically and industrially relevant ketones.

## Graphical abstract

Mechanochemically generated organocalcium species display unusual chemoselectivity toward carboxylates, enabling direct ketone synthesis without overaddition. Ball milling activates commercial calcium metal under ambient, solvent‐minimized conditions, providing a broad substrate scope, high functional‐group tolerance, and a practical one‐step conversion of carboxylic acids into valuable ketones.

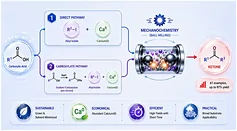

## 1 Introduction

As the third most abundant metallic element in the Earth's crust, calcium is inexpensive, non‐toxic, and environmentally benign. Systematic studies by Westerhausen established the fundamental synthetic methods, structures, and reactivity patterns of organocalcium compounds, revealing that these “heavy Grignard reagents” exhibit properties distinct from those of organolithium and organomagnesium reagents [1, 2, 3, 4, 5, 6]. Recent advances, including the development of dibenzylcalcium derivatives by Harder and coworkers [7, 8, 9, 10] and the structural authentication of dimethylcalcium by Anwander and coworkers [11] have further highlighted the unique opportunities offered by calcium‐based organometallics in synthesis and catalysis (Figure 1a) [12, 13, 14]. Despite these progresses, the practical use of organocalcium reagents remains largely underexplored, primarily due to the high atomization enthalpy of calcium and the challenges associated with the efficient pre‐activation of bulk calcium metal for organocalcium formation. As a result, organocalcium chemistry has long been underutilized compared to that of magnesium or lithium.

**FIGURE 1:**
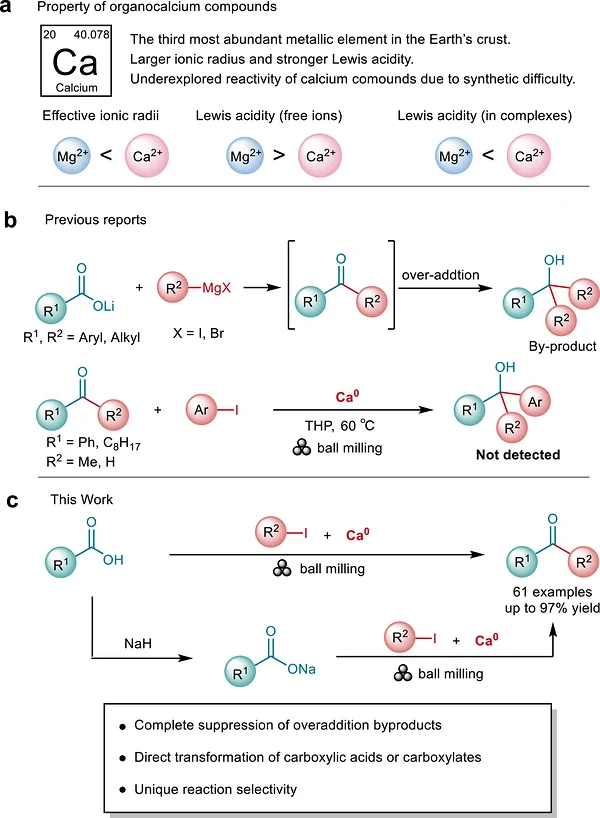
Synthesis of ketones. (a) Property of organocalcium compounds. (b) Reaction of in situ generated organocalcium reagents with various electrophiles. (c) This work: Ketone synthesis using in situ generated organocalcium reagents with carboxylic acids or carboxylate salts.

In recent years, advances in mechanochemical techniques have revitalized the direct activation of main‐group metals for the in situ generation of organometallic reagents [15, 16, 17, 18, 19, 20, 21, 22, 23, 24, 25]. Notably, a major breakthrough in organocalcium synthesis was reported by Ito, Kubota, and coworkers, who developed a ball‐milling strategy for the mechanochemical generation of organocalcium reagents [26]. This approach enables the direct coupling of aryl and primary alkyl iodides through mechano‐activation of organic iodides with calcium metal, providing a powerful platform for probing the distinctive reactivity of organocalcium species.

In contrast to organolithium and Grignard reagents, which readily undergo nucleophilic addition to aldehydes and ketones [27, 28], previous mechanochemical studies showed that in situ generated organocalcium species did not efficiently add to simple carbonyl compounds under the reported conditions [26] (Figure 1b). This distinct reactivity profile prompted us to explore whether organocalcium reagents could enable highly selective ketone synthesis from carboxylic acid derivatives, a transformation that is generally challenging with conventional organolithium or Grignard reagents due to competing overaddition.

Ketone synthesis from carboxylic acid derivatives is classically achieved using preactivated electrophiles such as acid chlorides or amides. However, these approaches often require highly reactive coupling partners, which may involve multistep preparation and the use of toxic or corrosive reagents such as thionyl chloride [29, 30]. Alternative methods, including transition‐metal‐catalyzed carbonylative cross‐coupling reactions, have been developed to overcome these limitations [31]. In addition, Zhang, Szostak, and coworkers recently reported the first mechanochemical synthesis of ketones from amides and boronic acids, in which overaddition was avoided through relative differentiation of the reactive groups [32, 33]. Inspired by these advances, we envisioned that mechanochemically generated organocalcium reagents could provide a complementary platform for direct and chemoselective ketone synthesis from carboxylate salts or carboxylic acids.

Herein, we disclose the first reaction report of organocalcium species with carboxylates or carboxylic acids to give ketone products, without over‐addition byproducts (Figure 1c). This unusual behavior suggests that organocalcium species preferentially engage hard, anionic, and chelating electrophiles, rather than neutral *π*‐electrophiles such as aldehydes and ketones. Meanwhile, coordination of the resulting geminal diol‐like tetrahedral intermediates to the calcium cation is proposed to suppress over‐addition side reactions [32]. This strategy would enable the direct use of carboxylate and organic iodide feedstocks for ketone synthesis without using toxic reagents [29, 30, 31, 34, 35, 36, 37], and pre‐formed, sensitive organometallics [15, 28, 38, 39, 40, 41, 42, 43, 44, 45, 46, 47, 48, 49, 50, 51], while offering improved substrate generality, functional‐group tolerance, step‐economy, and scalability compared to metallic lithium [52] and strontium [53] mediated processes in solution.

## 2 Results and Discussion

Due to the propensity of the acidic proton in carboxylic acids to quench organometallic reagents, the synthesis of ketones using organometallic compounds typically involves the prior conversion of carboxylic acids to their corresponding carboxylate salts. Among them, sodium carboxylates can be conveniently obtained in quantitative yields by treating various carboxylic acids with sodium hydride (NaH). As a model substrate, sodium benzoate (**2a**) was selected for the optimization of reaction conditions. In a nitrogen‐filled glovebox, **2a** (0.2 mmol), commercially available calcium granules (1.5 equiv relative to **2a**), *p*‐iodotoluene (2.0 equiv relative to **2a**), and two stainless‐steel ball (diameter 6 mm) were placed into a 1.5 mL stainless‐steel milling jar. The reaction was performed using a Retsch MM400 ball mill at 30 Hz for 60 min. Upon completion, the mixture was quenched with 1.0 M aq. HCl. The reaction progress was evaluated by monitoring the formation of ketone **4a**, the expected product derived from in situ generated organocalcium reagents. No formation of **4a** was detected in the absence of liquid‐assisted grinding (LAG). Analysis of the post‐reaction mixture revealed the complete recovery of the *p‐*iodotoluene starting material, indicating that the presence of LAG plays a crucial role in the generation of organocalcium species. (Figure 2a, entry 1). Ether additives are reported to improve the thermodynamic stability of organocalcium intermediates by coordinating with the calcium center [6]. Accordingly, we examined the effect of tetrahydrofuran (THF, 4.0 equiv relative to **2a**) as a LAG additive. This modification led to a substantial improvement in the yield of **4a**, reaching 90% (Figure 2a, Entry 2).

**FIGURE 2:**
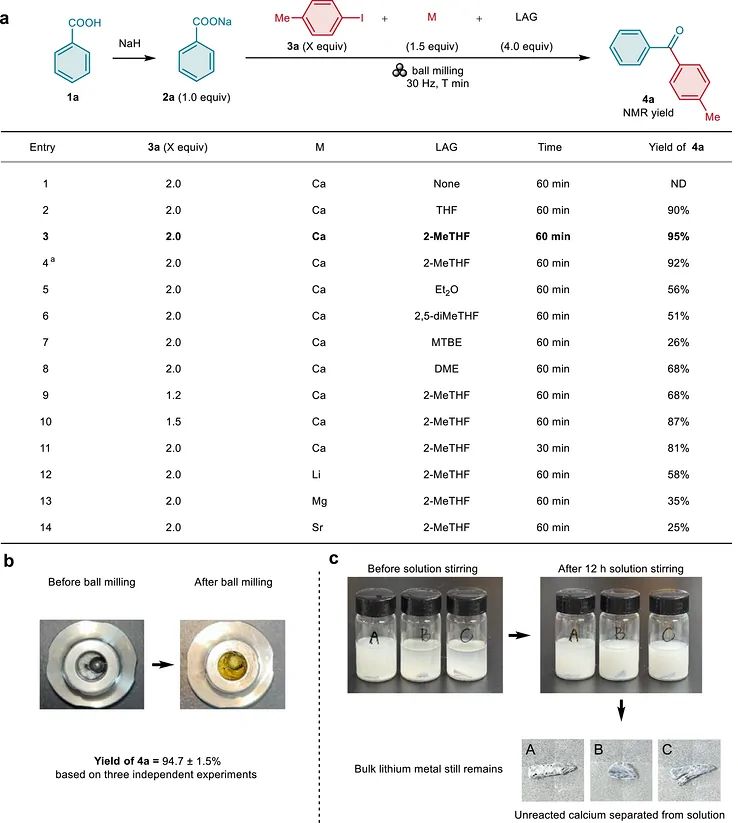
Reaction of carboxylate salts with in situ generated organocalcium reagents. (a) Optimization of mechanochemical reaction conditions. **2a** (0.2 mmol), **3a** (0.4 mmol), Ca (granule, 0.3 mmol) in a stainless‐steel ball‐milling jar (1.5 mL) with two stainless steel balls (6 mm), THF, tetrahydrofuran; 2‐MeTHF, 2‐methyltetrahydrofuran; 2,5‐diMeTHF, 2,5‐dimethyltetrahydrofuran; MTBE, *tert*‐butyl methyl ether; DME, dimethoxyethane. Integrated ^1^H NMR spectroscopic yields are shown. Added in air. ND: Not detected. (b) Reaction mixture before and after the reaction under mechanochemical conditions. **2a** (0.2 mmol), **3a** (0.4 mmol), Ca (granule, 0.3 mmol) in a stainless‐steel ball‐milling jar (1.5 mL) with two stainless steel balls (6 mm). (c) Reaction mixture before and after the reaction under solution conditions. **2a** (0.2 mmol), **3a** (0.4 mmol), Ca (granule, 0.3 mmol), and 2‐MeTHF (2 mL), added in N_2_ glovebox. ND: Not detected.

Encouraged by this result, we further explored a variety of ether solvents, including 2‐methyltetrahydrofuran (2‐MeTHF, Figure 2a, Entry 3), diethyl ether (Figure 2a, Entry 5), 2,5‐dimethyltetrahydrofuran (2,5‐diMeTHF, Figure 2a, Entry 6), methyl *tert‐*butyl ether (MTBE, Figure 2a, Entry 7), and 1,2‐dimethoxyethane (DME, Figure 2a, Entry 8). Among them, 2‐MeTHF gave the highest yield of **4a** (95%), surpassing THF, while other ether additives led to diminished yields. Remarkably, only a slight decrease in yield was observed when the reaction was performed under ambient air (Figure 2a, Entry 4), indicating a high tolerance of the system to moisture and oxygen. When the loading of calcium was reduced to 1.2 or 1.5 equivalents, the yields dropped to 68% (Figure 2a, Entry 9), and 87% (Figure 2a, Entry 10), respectively. This result suggests that an excess amount of calcium is essential to ensure complete metalation and efficient ketone formation, possibly due to the surface passivation of calcium. Shortening the reaction time to 30 min also led to a reduced yield (81%, Figure 2a, Entry 11). In addition, we compared the effects of milling balls with different weights and materials on the reaction yield and found that 6 mm stainless‐steel balls were the most suitable (Table S1).

Importantly, under all tested conditions, no overreaction leading to tertiary alcohol byproducts was observed, underscoring the high chemoselectivity of this mechanochemical transformation. This feature distinguishes the current protocol from conventional approaches with preformed organolithium or Grignard reagents under solution conditions, which often suffer from over addition issues in ketone synthesis [39]. Replacing calcium with lithium metal in the reaction with carboxylate salts led to a significant decrease in product yield (58%, Figure 2a, Entry 12). Similarly, when magnesium or strontium turnings were used in place of calcium, the yield dropped to 35% and 25%, respectively (Figure 2a, Entries 13 and 14). These observations suggest that lithium, magnesium, and strontium exhibit lower efficiency than calcium under the present conditions. This trend is further supported by DFT calculations. The activation barrier for nucleophilic addition of the organomagnesium species to the carboxylate carbonyl is substantially higher than that of the corresponding organocalcium species (33.9 vs. 22.3 kcal/mol), suggesting that the calcium pathway is kinetically more favorable (Table S2). In addition, the calculations indicate that the nucleophilic addition step generates a stable calcium‐coordinated tetrahedral intermediate. This intermediate does not readily collapse to a free ketone before acidic quenching, which is consistent with the absence of a ketone carbonyl signal in the pre‐quench ^13^C NMR spectrum (Figure S3). These results support the proposed mechanism involving a calcium‐bound geminal diolate‐like intermediate and provide a plausible rationale for the suppression of overaddition.

To evaluate the necessity of mechanochemical activation, we conducted solution conditions as comparative experiments. In nitrogen‐filled glovebox, a Schlenk tube was charged with **2a** (0.2 mmol), **3a** (0.4 mmol), and calcium granules (0.3 mmol), followed by the addition of 2‐MeTHF (2 mL). The mixture was stirred at room temperature using a PTFE‐coated magnetic stir bar. After 1 h of stirring, no visible change in the reaction solution was observed, and no product formation was detected by thin‐layer chromatography (TLC). Extending the reaction time to 12 h, only a trace of product **4a** was detected by TLC, and visible amounts of unreacted calcium metal were still present in the reaction mixture (Figure 2c). Considering that the surface condition of the metal may have a significant impact on the reaction rate, we repeated the solution experiment two more times under identical conditions and got similar results. In contrast, mechanochemical conditions yielded a consistent light‐yellow paste after 60 min of ball milling, with excellent reproducibility confirmed by three independent trials that consistently produced high yields and minimal variation (Figure 2b). This stark difference underscores the advantage of mechanochemistry in promoting surface‐dependent transformations, especially those involving heterogeneous activation of metallic reagents. The mechanical energy imparted during ball milling likely facilitates continuous surface renewal of the metal, thereby maintaining its reactivity throughout the course of the reaction. Based on these results, we established the optimized mechanochemical conditions as follows: sodium carboxylate (1.0 equiv), calcium metal (1.5 equiv), aryl iodide (2.0 equiv), and 2‐MeTHF (4.0 equiv), milled at 30 Hz for 60 min.

To evaluate the generality and functional group tolerance of this method, a diverse set of iodides was examined under the optimized conditions, using sodium benzoate (**2a**), calcium metal (1.5 equiv), and 2‐MeTHF (4.0 equiv) under ball milling at 30 Hz for 60 min (Figure 3a). The transformation proceeded smoothly with a wide range of substrates, affording the corresponding ketones in moderate to excellent isolated yields. Alkyl‐substituted aryl iodides, including sterically hindered examples such as 2‐iodotoluene, delivered the desired products in high yields (**4a**–**4e**, 78%–88%). Electron‐withdrawing substituents such as halogens (**4f**, 86%; **4** **g**, 70%; **4k**, 97%), trifluoromethyl (**4** **h**, 85%), and trifluoromethoxy (**4i**, 61%; **4j**, 58%) were well tolerated, indicating good chemoselectivity and potential for further derivatization. Similarly, electron‐donating groups such as methoxy, methylthio, and dimethylamino groups (**4l**–**4o**, 78%–95%) did not adversely affect the reaction efficiency. Substrates bearing sulfur‐ or oxygen‐containing heterocycles were also compatible with the reaction conditions, affording the corresponding ketones in good to excellent yields (**4u**–**4y**, 60%–90%). In contrast, nitrogen‐containing heterocycles gave lower yields, which can be attributed primarily to the formation of undesired side products.

**FIGURE 3:**
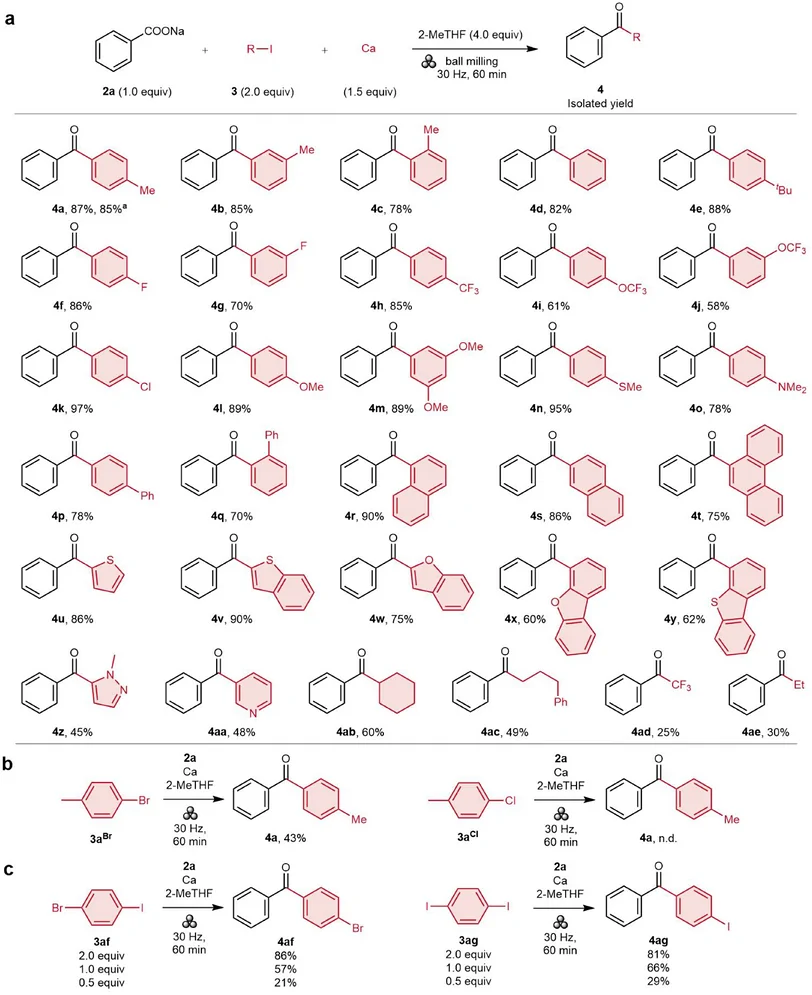
Reaction of organic halides. (a) Substrate scope of organic iodide compounds. Conditions: **2a** (0.2 mmol), **3** (0.4 mmol), Ca (0.3 mmol), and 2‐MeTHF (0.8 mmol) in a stainless‐steel ball‐milling jar (1.5 mL) with two stainless steel balls (6 mm). Isolated yields are shown. 2‐MeTHF, 2‐methyltetrahydrofuran. **2a** 2.0 mmol scale. (b) Reactivity of aryl chloride and aryl bromide. Conditions: **2a** (0.2 mmol), **3a** ^Br^ or **3a** ^Cl^ (0.4 mmol), Ca (0.3 mmol), and 2‐MeTHF (0.8 mmol). Isolated yields are shown. (c) Reaction selectivity of di‐halide substrates. Conditions: **2a** (0.2 mmol), **3af** or **3ag** (0.1 mmol or 0.2 mmol or 0.4 mmol), Ca (0.3 mmol) and 2‐MeTHF (0.8 mmol). Isolated yields (based on **2a**) are shown. n.d.: not detected.

The in situ generation of alkylcalcium species under mechanochemical conditions typically leads to homo‐coupling products as the major outcome, most likely via Wurtz‐type coupling [20], which has limited their utility in further derivatization reactions. However, in this study, we found that the reaction between alkylcalcium intermediates and carboxylate salts proceeds with sufficient rate to effectively compete with the homo‐coupling pathway, thereby enabling the formation of the desired ketone products (**4ab**–**4ae**, 25%–60%).

We also compared the reactivity of aryl bromides and aryl chlorides under the standard mechanochemical conditions. When 4‐bromotoluene (**3a^Br^**) was used as substrate, the yield of product **4a** decreased to 43%, and a significant amount of unreacted carboxylate salt **2a** was detected, indicating incomplete conversion. In the case of 4‐chlorotoluene (**3a^Cl^**), no reaction was observed (Figure 3b). These results suggesting that the rate of calcium insertion into the C─I bond is significantly higher than that into the C─Br bond, while insertion into the C─Cl bond does not occur under these conditions. Furthermore, we explored the reactivity of substrates bearing multiple carbon–halogen bonds. When 4‐bromoiodobenzene (**3af**) was used, calcium selectively inserted into the C─I bond without affecting the C─Br bond, affording the desired ketone product **4af**. In the case of 1,4‐diiodobenzene, the reaction occurred selectively at only one of the two C─I bonds, and the mono‐functionalized product **4ag** was obtained even when the equivalent of the alkyl iodide (**3ag**) was reduced to 0.5 equiv (one‐third of calcium metal, Figure 3c). These results clearly indicate that in polyhalogenated substrates, calcium insertion takes place at a single site, enabling the preservation of other carbon─halogen bonds. This reaction selectivity highlights the unique reactivity of organocalcium reagents, renders the method particularly attractive for further functionalization or derivatization strategies.

We further explored the scope of carboxylate salts as electrophile under the optimized mechanochemical conditions (Figure 4a). The carboxylate salts used in this study were either commercially available or readily prepared by deprotonation of the corresponding carboxylic acids with sodium hydride (see Section S2 for details). A wide variety of aryl carboxylates bearing alkyl, electron‐withdrawing, or electron‐donating substituents reacted smoothly to afford the corresponding ketones in moderate to excellent yields (**4ah**–**4bb**, 41%–65%), demonstrating broad functional group tolerance. In addition, both linear and cyclic aliphatic carboxylates were viable substrates, providing the desired ketone products in moderate yields (**4bc**–**4be**, 42%–62%). Notably, the reaction also tolerated alkynyl‐substituted carboxylates, albeit with relatively low efficiency. Moreover, the influence of the carboxylate counterion was investigated (Figure 4b). Among the alkali metal salts tested, sodium carboxylates consistently gave the highest yields, followed by potassium salts, while lithium carboxylates showed the lowest reactivity. These results suggest that the choice of counterion can significantly impact the efficiency of the reaction, possibly due to differences in solubility or aggregation state under mechanochemical conditions.

**FIGURE 4:**
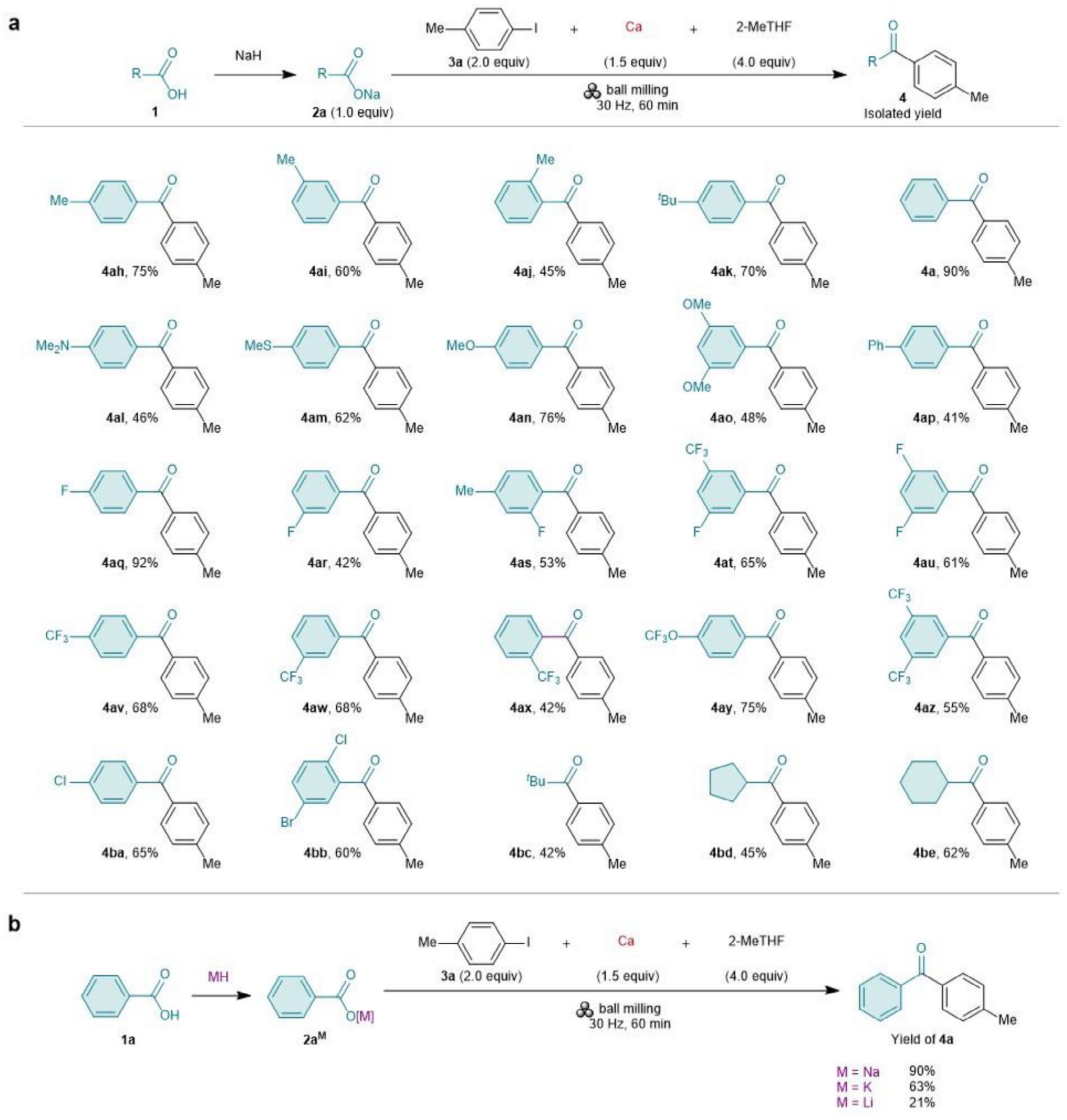
Reaction of carboxylic acid salts. (a) Substrate scope of carboxylic acids. Conditions: **2** (0.2 mmol), **3a** (0.4 mmol), Ca (0.3 mmol), and 2‐MeTHF (0.8 mmol) in a stainless‐steel ball‐milling jar (1.5 mL) with two stainless steel balls (6  mm). Isolated yields are shown. 2‐MeTHF, 2‐methyltetrahydrofuran. (b) Influence in reactivity of different metals. Conditions: **2a^Na^**, **2a^Li^** or **2a^K^** (0.2 mmol), **3a** (0.4 mmol), Ca (0.3 mmol), and 2‐MeTHF (0.8 mmol). Isolated yields are shown.

To further streamline the reaction protocol, we examined the direct use of carboxylic acids as substrates under mechanochemical conditions. A mixture of aryl or alkyl carboxylic acid (0.2 mmol), aryl iodide (3.0 equiv), calcium metal (3.0 equiv), and 2‐MeTHF (4.0 equiv) was subjected to ball milling at 30 Hz for 60 min. After workup, the corresponding ketone products were obtained in moderate yields (Figure 5, 49%–66%). This one‐step protocol eliminates the need for prior preparation of carboxylate salts and demonstrates the practicality of the mechanochemical approach for rapid ketone synthesis directly from readily available carboxylic acids.

**FIGURE 5:**
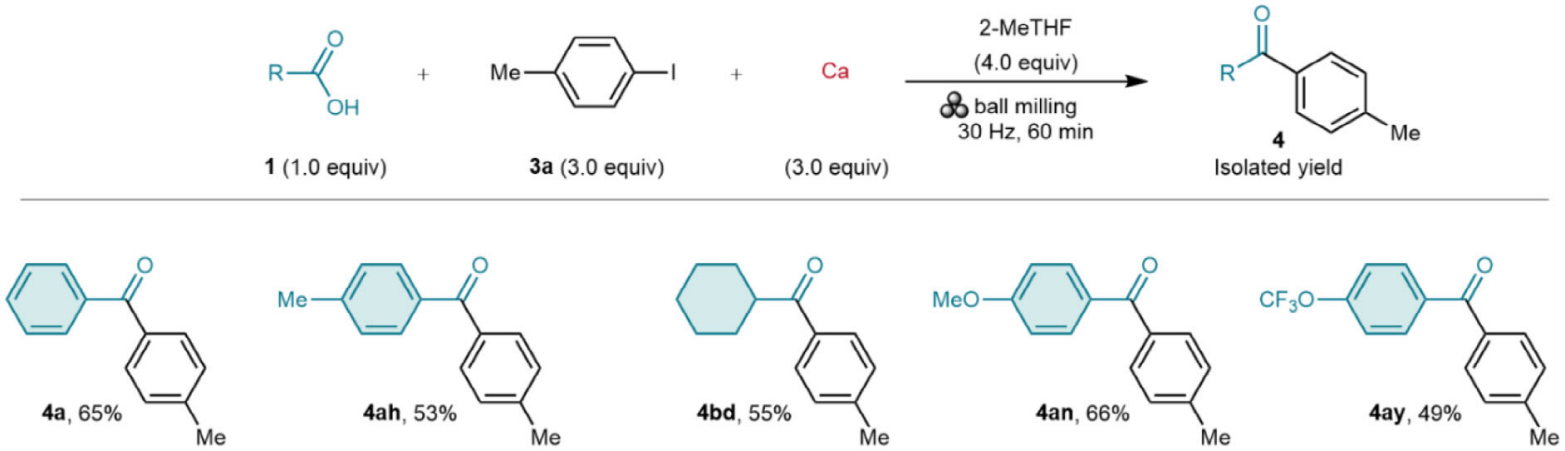
Direct reaction of carboxylic acids. Conditions: **1** (0.2 mmol), **3a** (0.6 mmol), Ca (0.6 mmol), and 2‐MeTHF (0.8 mmol) in a stainless‐steel ball‐milling jar (1.5 mL) with two stainless steel balls (6 mm). Isolated yields are shown.

Finally, we demonstrated the synthetic utility of this mechanochemical protocol by applying it to the preparation of several pharmaceutically and industrially relevant ketones (Figure 6). 4,4′‐Difluorobenzophenone (DFBP, **4bf**) is a key intermediate in the synthesis of the antihistamine drug flunarizine and serves as a major monomer to produce polyether ether ketone (PEEK). The current industrial route to DFBP typically involves diazotization of 4,4′‐diaminodiphenylmethane with sodium nitrite in hydrofluoric acid, followed by oxidative conversion to the ketone, with a reported yield of approximately 80% [54, 55]. However, this process suffers from serious drawbacks, including the use of highly corrosive and toxic reagents, environmental concerns, and the potential risk of explosion associated with diazonium salts. In contrast, our mechanochemical approach using sodium carboxylate **2bf** and aryl iodide **3bf** as substrates furnished DFBP in 85% yield, without the need for hazardous chemicals and with minimal waste generation.

**FIGURE 6:**
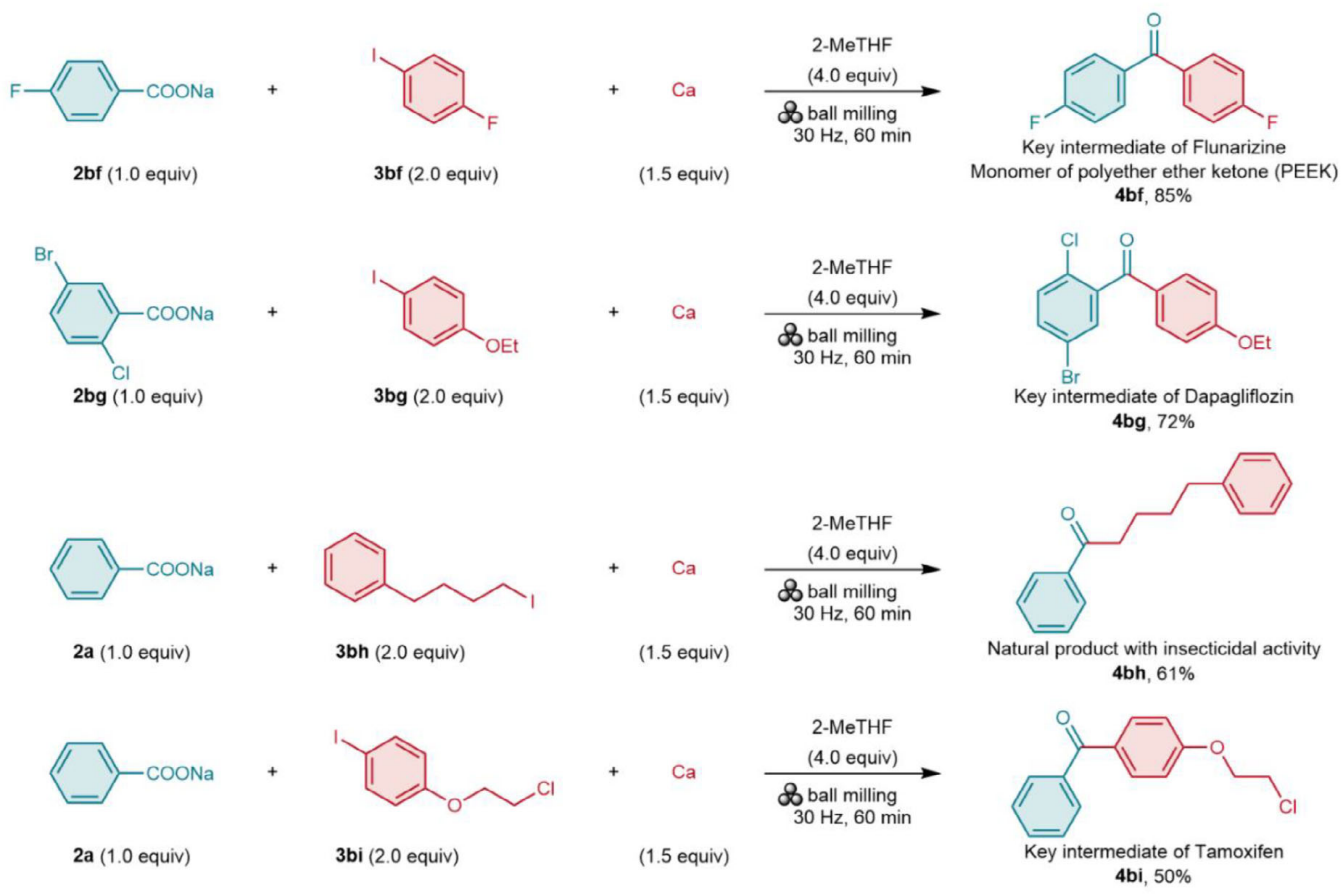
Application in the synthesis of commercially used ketone compounds. Conditions: 2 (0.2 mmol), 3 (0.4 mmol), Ca (0.3 mmol), and 2‐MeTHF (0.8 mmol) in a stainless‐steel ball‐milling jar (1.5 mL) with two stainless steel balls (6 mm). Isolated yields are shown.

Dapagliflozin, the first SGLT2 inhibitor approved for the treatment of Type 2 diabetes, also contains a diaryl ketone core [56, 57]. A key intermediate in its synthesis, 5‐bromo‐2‐chloro‐4′‐ethoxybenzophenone (**4bg**), is traditionally prepared via a Friedel–Crafts acylation of an aryl substrate with the corresponding acid chloride derived from 5‐bromo‐2‐chlorobenzoic acid using oxalyl chloride. This approach requires toxic reagents and labor‐intensive steps. By employing carboxylate **2bg** and iodide **3bg** under our standard ball‐milling conditions, **4bg** was obtained in 72% yield in a single step, significantly simplifying the synthetic route and avoiding the use of hazardous reagents.

Moreover, this protocol was successfully applied to the synthesis of a natural product (**4bh**, 61%) with reported insecticidal activity [58], as well as a key intermediate (**4bi**, 50%) in the preparation of tamoxifen [59], a widely used selective estrogen receptor modulator. All of these transformations were accomplished directly from carboxylate salts in a single step, highlighting the practicality and broad applicability of the method. Compared to conventional solution‐phase approaches, the mechanochemical strategy not only avoids the use of toxic reagents but also significantly reduces solvent usage and waste generation, making it a greener and more sustainable alternative for ketone synthesis.

## 3 Conclusion

In summary, we have developed a practical and chemoselective mechanochemical protocol for the synthesis of ketones via the reaction of in situ generated organocalcium reagents with carboxylate salts or carboxylic acids. The method proceeds under solvent‐minimized conditions, displays excellent reproducibility, and tolerates a wide range of functional groups and heterocycles. The operational simplicity and reproducibility position this method as a valuable synthetic tool for the efficient construction of structurally diverse ketones. Contrast studies highlight the superiority of calcium over other metals in terms of chemoselectivity and reactivity under mechanochemical conditions. Furthermore, this strategy has been successfully applied to the synthesis of pharmaceutically and industrially valuable ketones, demonstrating its potential as a greener and operationally simple alternative to conventional organometallic ketonization protocols.

## Author Contributions

**Xiaoliang Zheng**: project administration, writing – review and editing, supervision. **Zekun Yang**: conceptualization, validation, supervision, writing – original draft. **Mengyao Pei**: methodology, investigation, formal analysis, writing – original draft. **Jiemin Wang**: investigation, methodology. **Yufang Yang**: methodology, investigation. **Peile Ma**: methodology, investigation. **Xiaofeng Wei**: supervision, writing – review and editing, conceptualization. **Yangyang Shen**: investigation, writing – review and editing. **Xueyan Yang**: methodology, investigation. **Zezhu Li**: investigation, writing – review and editing.

## Funding

We acknowledge the financial support from the Basic Research Program of Jiangsu (No: BK20250462 to Z. Y.), the Scientific Research Foundation of Wuhan Institute of Technology (K2025106 to Z. Y.), the High‐Level Introduction Plan of Shaanxi Province (to X. W.), the start‐up funds from Xi'an Jiaotong University (to X. W.), the National Natural Science Foundation of China (22571243 to X. W), and the Fundamental Research Funds for the Central Universities (xtr052025012 to X. W), Joint project between the Science and Technology Department of the State Administration of Traditional Chinese Medicine and Zhejiang Province (GZY‐KJS‐ZJ‐2026‐086 to X. Z.), Zhejiang Provincial Special Fund for Supporting Provincial‐Level Scientific Research Institutes (YS202603 to X. Z.).

## Conflicts of Interest

The authors declare no conflicts of interest.

## Supporting information




**Supporting File**: advs76440‐sup‐0001‐SuppMat.docx.

## Data Availability

The data that support the findings of this study are available in the Supporting Information of this article.
