# Supplementary material for: Calcium‐Mechanochemistry Enabled Ketone Synthesis From Organic Iodides and Carboxylic Acids
Source: Adv Sci (Weinh). 2026 Jul 9;13(54):e76440. doi: 10.1002/advs.76440 (PMC13348655; doi:10.1002/advs.76440)
Supplement: Supplementary file 1 [file ADVS-13-e76440-s001.docx]

**Supplementary information**

**Calcium-mechanochemistry enabled ketone synthesis from organic iodides and carboxylic acids**

Mengyao Pei^1†^, Ze-Kun Yang^2,3†*^, Xueyan Yang^4^, Yufang Yang^1^, Yangyang Shen^5^, Jiemin Wang^1^, Peile Ma^1^, Zezhu Li^7^, Xiaoliang Zheng^6*^, Xiaofeng Wei ^1*^

^1^ School of Pharmacy, Xi’an Jiaotong University, No.76, Yanta West Road, Xi’an, 710061, China.

^2^ School of Chemical Engineering and Pharmacy, Pharmaceutical Research Institute, Wuhan Institute of Technology, Wuhan, 430205, China.

^3^ Xi'an Jiaotong University Suzhou Academy, Suzhou, 215123, China.

^4^ Department of Pharmacy, The First Affiliated Hospital of Xi’an Jiaotong University, Xi’an, Shaanxi, 710061, China.

^5^ Frontier Institute of Science and Technology, Xi'an Jiaotong University, Xi’an, China.

^6^ Zhejiang Key Laboratory of Tumor Molecular Diagnosis and Individualized Medicine, School of Basic Sciences and Forensic Medicine, Hangzhou Medical College, Hangzhou, Zhejiang, 310014, China.

^7^ Department of Chemistry, University College London, Gower Street, London, WC1E 6BT, UK.

†These authors contributed equally to this work.

E-mail: [xiaofeng.wei@xjtu.edu.cn](mailto:xiaofeng.wei@xjtu.edu.cn), zekun.yang@wit.edu.cn, [zhengxl@hmc.edu.cn](mailto:guohui@xjtufh.edu.cn)

Table of contents

1. General information about chemicals and instrumentation	S3

2. Synthesis of Substrates	S5

3. General procedure of mechanochemical reactions	S7

4. Comparative experiments under solution conditions	S9

5.In-situ generated organocalcium species with carbonyl compounds	S10

6. Comparison of different milling balls	S11

7. Comparison between aryl iodide and alkyl iodide	S11

8. NMR analysis of the pre-quench mixture	S12

9. Computational Details	S13

10. Characterization of ketone products	S23

11. Application expansion	S45

12. References	S48

13. NMR Spectra	S51

14. Substrates incompatible with this reaction	S132

**1. General information about chemicals and instrumentation**

Unless otherwise specified, all reactions were carried out in dry solvents under a nitrogen or argon atmosphere. Commercial reagents were obtained from Adamas, Aladdin, Alfa Aesar, Bidepharm, Leyan, and TCI, and used as received without further purification. Reaction progress was monitored by thin-layer chromatography (TLC) using Silicycle 200 mm silica gel GF-254 plates, visualized under UV light at 254 nm. Products were purified by flash column chromatography using silica gel (200–300 mesh) or by preparative thin-layer chromatography (PTLC). The specification of the thin-layer plate used in PTLC is PLC Silica Gel 60 GF254, purchased from Leyan.

Mechanochemical reactions were performed in stainless steel grinding jars with stainless steel balls using a Retsch MM 400 ball mill **(Figure S1)**.

**Fig.S1.** Retsch MM400 (left), stainless jar (1.5 mL) and ball (6.0 mm diameter) (right) used in this study.

NMR spectra were recorded on JEOL 400 MHz or Bruker 600 MHz spectrometers. Fluorobenzene was employed as an internal standard to determine NMR yields. Signal multiplicities are reported as follows: s = singlet, d = doublet, t = triplet, q = quartet, and m = multiplet.

High-resolution mass spectra were acquired using electrospray ionization (ESI) or electron impact (EI) techniques on a WATERS I-Class VION IMS QToF double-focusing magnetic sector mass spectrometer. All analytical measurements were conducted at the Instrument Analysis Center of Xi’an Jiaotong University (XJTU).

**Safety Considerations.** Although the reaction can be charged under ambient atmosphere, it is still recommended that calcium metal be stored and all reaction preparations be carried out in a glovebox to minimize exposure to air and moisture. After the reaction, the mixture typically contains residual calcium particles, which should be quenched with great caution. A slow, portionwise addition of a suitable alcohol (e.g., *i*-PrOH or EtOH) at low temperature is advised to avoid rapid hydrogen evolution and potential exothermic events. Direct quenching with water should be strictly avoided due to the risk of rapid hydrogen evolution.

For mechanochemical reactions, mechanochemical equipment must be inspected to ensure proper sealing of jars, correct seating of grinding balls, and the absence of metal fatigue. Overfilling of jars and operation outside the recommended frequency range should be avoided to prevent mechanical failure [1].

**2. Synthesis of Substrates**

**General procedure-1: Synthesis of carboxylate salt substrates**

Sodium hydride (60%, dispersion in paraffin liquid, 200 mg, 5.0 mmol) and anhydrous THF (50 mL, 1.0M) were added to a round-bottom flask under a nitrogen atmosphere and stirred at room temperature for 10 mins. **1a** (610 mg, 5.0 mmol) was then added, and the reaction mixture was stirred for 6 h. After completion of the reaction, the mixture was extracted with dichloromethane and water four times. The combined aqueous layers were then concentrated under reduced pressure to afford the target compound.

**General procedure-2** [2]**: Synthesis of (4-Iodobutyl)benzene (3bh)**

To the solution of PPh_3_ (1.9 g, 7.5 mmol), I_2_ (1.9 g, 7.5 mmol), and imidazole (750 mg, 11 mmol) in CH_2_Cl_2_ (20 mL), was slowly added 4-phenylbutan-1-ol ( 765 uL, 5 mmol) at room temperature. The reaction mixture was stirred for 2 h. After quenching the reaction by the addition of saturated aqueous Na_2_S_2_O_3_ (100 mL), the additional CH_2_Cl_2_ (50 mL) and brine (100 mL) were added and separated. The aqueous phase was extracted by CH_2_Cl_2_ (50 mL × 2), and the combined organic extract was washed by saturated aqueous NH_4_Cl (100 mL), followed by dried over MgSO_4_. After the filtration and the concentration under reduced pressure, the crude product thus obtained was purified by the flash chromatography (Hexane/AcOEt = 3:1) to give **3bh** (1.2 g, 4.5 mmol, 90% yield). **^1^H NMR** (400 MHz, CDCl_3_) δ 7.29 (t, *J* = 7.5 Hz, 2H), 7.19 (t, *J* = 8.5 Hz, 3H), 3.21 (t, *J* = 6.9 Hz, 2H), 2.64 (t, *J* = 7.5 Hz, 2H), 1.88 (dd, *J* = 14.1, 7.6 Hz, 2H), 1.75 (q, *J* = 7.9 Hz, 2H). **^13^C NMR** (101 MHz, CDCl_3_) δ 141.8, 128.4, 125.9, 34.8, 32.9, 32.2, 6.7.

**General procedure-3** [3]**: Synthesis of 1-(2-Chloroethoxy)-4-iodobenzene (3bi)**

To a solution of 4-iodophenol (660 mg, 3.0 mmol) and tetrabutylammonium hydrogensulfate (16.7 mg, 0.05 mmol) in 1,2-dichloroethane (3.4 mL, 0.9 mmol) was added 3 mol/L aqueous sodium hydroxide (1.7 mL) and the mixture was refluxed for 1h after which 6 mol/L aqueous sodium hydroxide (315 uL) was added. The resulting solution was refluxed for 24 h and cooled to room temperature. The organic layer was separated, diluted with petroleum ether and extracted with saturated aqueous ammonium chloride. The organic layer was dried with anhydrous MgSO_4_ and concentrated under reduced pressure. The crude product was purified by column chormatography (Hexane : Ethyl acetate, 9:1) affording **3bi** (716 mg, 2.55 mmol) in 85% yield as a white solid. **^1^H NMR** (400 MHz, CDCl_3_) δ 7.57 (d, *J* = 8.8 Hz, 2H), 6.69 (d, *J* = 2.1 Hz, 2H), 4.19 (t, *J* = 5.8 Hz, 2H), 3.80 (t, *J* = 5.9 Hz, 2H). **^13^C NMR** (101 MHz, CDCl_3_) δ 158.1, 138.4, 117.1, 83.6, 68.1, 41.7.

**3. General procedure of mechanochemical reactions**

**General procedure-4: Reaction of *in situ* generated organocalcium reagents with carboxylate salts.**

**2a** (0.2 mmol, 1.0 equiv), **3a** (0.4 mmol, 2 equiv), Ca (0.3 mmol, 1.5 equiv) and dry 2-CH_3_THF (0.8 mmol, 4.0 equiv) were added to a stainless-steel milling jar (1.5 mL) with two stainless steel balls (6.0 mm diameter) in glove box and the reaction was carried out in a Retsch MM400 through 30 Hz ball milling. After ball milling for 60 mins, the reaction mixture was quenched with dilute hydrochloric acid. The resulting mixture was then subjected to short-column chromatography on silica gel and eluted with dichloromethane. Removal of the solvent under reduced pressure afforded a residue, which was purified by preparative thin-layer chromatography (PTLC).

**General procedure-5: Reaction of *in situ* generated organocalcium reagents with carboxylic acids.**

**1a** (0.2 mmol, 1.0 equiv.), **3a** (0.6 mmol, 3 equiv), Ca (0.6 mmol, 3 equiv) and dry 2-CH_3_THF (0.8 mmol, 4.0 equiv) were added to a stainless-steel milling jar (1.5 mL) with two stainless steel balls (6.0 mm diameter) in glove box and the reaction was carried out in a Retsch MM400 through 30 Hz ball milling. After ball milling for 60 mins, the reaction mixture was quenched with dilute hydrochloric acid. The resulting mixture was then subjected to short-column chromatography on silica gel and eluted with dichloromethane. Removal of the solvent under reduced pressure afforded a residue, which was purified by preparative thin-layer chromatography (PTLC).

**General procedure-6: Verification of the formation of the Organocalcium Reagent**

**3cf** (0.4 mmol, 1.0 equiv), Ca (0.6 mmol, 0.75 equiv) and CD_3_OD (200 uL) were added to a stainless-steel milling jar (1.5 mL) with two stainless steel balls (6.0 mm diameter) in glove box and the reaction was carried out in a Retsch MM400 through 30 Hz ball milling. After ball milling for 60 mins, The resulting mixture was then subjected to short-column chromatography on silica gel and eluted with dichloromethane. Due to the difficulty in separating the deuterated product from the starting material, the reaction outcome was determined by NMR analysis of the mixture. The crude product was further purified by column chromatography on silica gel (hexane) to afford the mixture (70.8 mg, n**_3cf_** = 0.19 mmol, n**_4cf_** = 0.16 mmol) as a colorless oil. **^1^H NMR** (400 MHz, Methanol-*d*_4_) δ 7.60 (d, *J* = 8.3 Hz, 2H), 7.38 (d, *J* = 7.9 Hz, 2H), 7.26 (d, *J* = 7.9 Hz, 2H), 7.19 (d, *J* = 8.5 Hz, 2H), 1.31 (s, 9H), 1.29 (s, 9H).

**4.** **Comparative experiments under solution conditions**

**2a** (0.2 mmol, 1.0 equiv), **3a** (0.4 mmol, 2 equiv), Ca (0.3 mmol, 1.5 equiv) and dry 2-CH_3_THF (2.0 mL, 0.1 M) were added to round-bottom flask and the mixture was then stirred at room temperature for 12 hours. After the reaction was complete, the reaction mixture was quenched with dilute hydrochloric acid. The resulting mixture was then subjected to short-column chromatography on silica gel and eluted with dichloromethane. Removal of the solvent under reduced pressure afforded a residue, which was purified by preparative thin-layer chromatography (PTLC).

**S2a	S2b	S2c**

**S2d	S2e	S2f**

**Fig.S2. S2a** shows the reaction mixture under solution conditions, **S2b** shows the reaction mixture after stirring for 5 h, and **S2c** for 12 h. **S2d-S2f** represent the calcium used in reactions A, B, and C (m_A_=61.2 mg, m_B_=60.8 mg, m_C_=60.5 mg), respectively. The left images show the state of calcium before the reaction, while the right images show the state after the reaction. It can be observed that the calcium has noticeably reduced in size, but has not fully reacted.

5. **In-situ generated organocalcium species with carbonyl compounds**

**5a** or **6a** (0.2 mmol, 1.0 equiv), **3a** (0.4 mmol, 2 equiv), Ca (0.3 mmol, 1.5 equiv) and dry 2-CH_3_THF (2.0 mL, 0.1 M) were added to round-bottom flask and the mixture was then stirred at room temperature for 12 hours. After the reaction was complete, the reaction mixture was quenched with dilute hydrochloric acid. The resulting mixture was then analyzed by GC-MS and purified by preparative thin-layer chromatography (PTLC). Approximately 10% of the **3a** was recovered, and the carbonyl compounds **5a** and **6a** were recovered almost completely, and no detectable nucleophilic addition products or carbonyl reduction products were observed.

**6. Comparison of different milling balls**

**General procedure-4: Reaction of *in situ* generated organocalcium reagents with carboxylate salts.**

**Table S1.** Effect of milling ball material, diameter, and mass on the mechanochemical reaction efficiency.

| Entry | Milling ball material | Diameter (mm) | Mass per ball (mg) | Yield of 4a (%) *^a^* |
| --- | --- | --- | --- | --- |
| 1 | Zirconium oxide | 7 | 1115.6 | 80 |
| 2 | Tungsten carbide | 6 | 1683.7 | 78 |
| 3 | Polyoxymethylene (POM) | 6 | 157.2 | 56 |
| 4 | Stainless-steel | 3 | 112.0 | 75 |
| 5 | Stainless-steel | 4 | 265.7 | 78 |
| 6 | Stainless-steel | 6 | 893.8 | 91 |

*^a^* Yields were determined by ¹H NMR spectroscopy using an internal standard.

**7. Comparison between aryl iodide and alkyl iodide**

**General procedure-4: Reaction of *in situ* generated organocalcium reagents with carboxylate salts.**

Result analysis: 22.1 mg of **4a** was obtained, with an isolated yield of 56%, while no cyclohexyl phenyl ketone product was observed.

**8. NMR analysis of the pre-quench mixture**

After completion of the ball-milling reaction, the unquenched mixture was transferred under an inert atmosphere and treated with anhydrous C_6_D_6_. The resulting suspension was filtered, and the crude filtrate was directly analyzed by NMR spectroscopy. In the ^13^C NMR spectrum of this pre-quench mixture, no resonance was observed in the typical ketone carbonyl region of 190–210 ppm.

**Fig.S****3. ^13^C NMR**-spectrum (101 MHz, C_6_D_6_) of the pre-quench mixture.

**9. Computational Details**

The theoretical calculations were performed using the Gaussian 16 program [4] with PBE [5]functional. Geometries and frequency calculations were conducted using the Def2-SVP basis set, and the single-point energy refinements were further performed with Def2-TZVP basis set [6, 7]. Empirical dispersion corrections were included using the DFT-D3. The solvation effects were taken into account using the IEFPCM implicit solvent model with tetrahydrofuran as the solvent [8].

**Table S2.** The computed potential energies (E, a.u.) are at the PBE/Def2-TZVP//Def2-SVP level. The relative Gibbs energies (ΔG; at 298.15 K and 1 atm) are in kcal/mol.

| **Complex** | E | G | ΔG |
| --- | --- | --- | --- |
| **[PhCa(THF)I)_]2_** | -3342.336579 | -3341.792069 | 0 |
| **PhCOO^-^** | -420.0246279 | -419.9578069 | - |
| **IM1-Ca** | -1858.954916 | -1858.712669 | -11.44916183 |
| **TS1-Ca** | -1858.922713 | -1858.67712 | 10.85811063 |
| **IM2-Ca** | -1858.955044 | -1858.706116 | -7.337531334 |
|  |  |  |  |
| **IM1-Mg** | -1381.522325 | -1381.2752 | 0 |
| **TS1-Mg** | -1381.470858 | -1381.221136 | 33.92598736 |
| **IM2-Mg** | -1381.515344 | -1381.262166 | 8.178833321 |

**Fig. S4.** Free energy profile (kcal mol−1) for the reaction between organocalcium reagent and carboxylate salt.

**Fig. S5.** Free energy profile (kcal mol−1) for the key step in the reaction between Grignard reagent and carboxylate salt

**Cartesian coordinates of all stationary points optimized at PBE/Def2-SVP level.**

**[PhCa(THF)I)]_2_**

Coordinates (Angstroms)

X	Y	Z

Ca	2.00257400	-0.07469300	-0.74949300

C	0.50226900	2.02061200	-0.86188400

C	0.70011900	3.04647100	0.10702900

C	0.58998400	2.46256800	-2.21272000

C	0.96493000	4.38927600	-0.22754300

H	0.63836800	2.79769900	1.18452300

C	0.86060000	3.79779400	-2.57741500

H	0.44479400	1.72721900	-3.02926700

C	1.05013300	4.76859900	-1.57888200

H	1.10579000	5.14296800	0.56541900

H	0.91930600	4.08439000	-3.64105000

H	1.26011400	5.81545000	-1.85122900

Ca	-1.19684200	0.34010100	0.17132900

C	0.77339300	-0.99134100	1.27913800

C	1.15352700	-0.43974400	2.53527500

C	0.67085400	-2.41140300	1.26088400

C	1.42177300	-1.22414500	3.67640700

H	1.24574600	0.65808700	2.64305600

C	0.93192700	-3.21889500	2.38495500

H	0.37685200	-2.91348400	0.31913700

C	1.31455100	-2.62302900	3.60066900

H	1.71271700	-0.74485600	4.62610200

H	0.83837800	-4.31618100	2.31694000

H	1.52290300	-3.24622000	4.48522000

I	-0.03294300	-1.52742500	-2.61035100

I	-2.49718800	1.86167700	2.54686500

O	3.72834500	1.40884200	0.00010300

C	4.22492400	2.45880200	-0.88399300

C	3.98237300	1.77078900	1.38419000

C	4.70421700	3.58127800	0.03788500

H	5.05167800	2.03006000	-1.49082400

H	3.39909600	2.76986500	-1.55699400

C	5.08541900	2.82040700	1.31642800

H	3.04935200	2.18772400	1.82412500

H	4.25336600	0.84870800	1.93519900

H	3.87110600	4.28428600	0.24131900

H	5.54253300	4.15511000	-0.40159900

H	5.11154800	3.46194600	2.21823200

H	6.07756400	2.33452900	1.20745500

O	3.61485700	-1.84076100	-0.49369000

C	4.28445500	-2.01792900	0.79086600

C	3.58289900	-3.10446100	-1.21086800

C	4.76918200	-3.47056300	0.81275000

H	5.10720600	-1.27700700	0.86160000

H	3.54099200	-1.81312700	1.59130800

C	3.78251400	-4.16726000	-0.13682600

H	2.61150300	-3.16453900	-1.74343200

H	4.40651800	-3.11612300	-1.96018200

H	4.76219200	-3.89255100	1.83620700

H	5.80356800	-3.54453800	0.41821500

H	2.82312200	-4.36869600	0.38172700

H	4.16733600	-5.12006900	-0.54873500

O	-2.55136200	-1.59652200	0.62086000

C	-3.06642800	-2.58444900	-0.29095800

C	-2.83078900	-2.10647900	1.94230800

C	-4.40174400	-3.03346300	0.32754300

H	-3.14233300	-2.11249800	-1.28871800

H	-2.34032700	-3.42628500	-0.36008300

C	-4.24775700	-2.69164300	1.83687500

H	-2.07278800	-2.88276200	2.19295200

H	-2.72970100	-1.25868300	2.64800600

H	-4.58303000	-4.11069800	0.14783100

H	-5.24910700	-2.47594500	-0.11847500

H	-4.36789500	-3.57682600	2.49075000

H	-4.99971000	-1.94029900	2.14817300

O	-2.98449800	0.87662500	-1.32141700

C	-4.39212900	0.79518400	-1.01150600

C	-2.90200000	1.22818500	-2.71700000

C	-5.05742800	0.20735400	-2.27506600

H	-4.49328400	0.17523000	-0.10067100

H	-4.76945500	1.81685200	-0.78516400

C	-3.97835100	0.36476400	-3.38103600

H	-3.10708700	2.31732100	-2.83195400

H	-1.86976900	1.01183200	-3.05187200

H	-5.98864500	0.75505200	-2.51699700

H	-5.32971900	-0.85664700	-2.13110400

H	-4.37117300	0.83021400	-4.30538700

H	-3.54654600	-0.61919800	-3.65361700

**PhCOO-**

Coordinates (Angstroms)

X	Y	Z

O	2.38631600	-1.13635000	0.02833700

O	2.38624900	1.13637500	-0.02830900

C	0.28084600	-0.00001500	-0.00002700

C	-0.43954200	1.21106600	0.01061500

C	-0.43955500	-1.21108300	-0.01064700

C	-1.84418700	1.21621500	0.01272100

C	-1.84420200	-1.21621000	-0.01271400

C	-2.55173800	0.00000500	0.00001800

C	1.83324000	-0.00000900	-0.00000300

H	0.14702700	2.14355300	0.01751500

H	0.14696800	-2.14359800	-0.01759600

H	-2.39500900	2.17126000	0.02295100

H	-2.39504100	-2.17124600	-0.02291800

H	-3.65364100	0.00001100	0.00003400

**IM1-Ca**

Coordinates (Angstroms)

X	Y	Z

O	1.73261800	-0.15164000	1.02183100

O	1.68913100	-1.53200900	-0.74119900

C	3.83197200	-0.77635300	0.03728800

C	4.51861300	-1.51801900	-0.94503400

C	4.56385900	0.01458800	0.94599800

C	5.91868300	-1.47074600	-1.01781500

C	5.96396200	0.06378300	0.87333900

C	6.64350400	-0.67942000	-0.10859800

C	2.32400800	-0.82800800	0.11328400

H	3.92762500	-2.12758300	-1.64459400

H	4.00899300	0.58708200	1.70401700

H	6.45020700	-2.05220200	-1.78726800

H	6.53112800	0.68437500	1.58462700

H	7.74263900	-0.64116600	-0.16587500

Ca	-0.39170700	-0.63400600	0.01411200

C	-0.63491900	1.48760200	-1.23110300

C	0.39578000	2.46461400	-1.13111500

C	-1.81977300	1.95218900	-1.86697400

C	0.26892800	3.78477800	-1.61137300

H	1.35162700	2.19113400	-0.64006000

C	-1.97921000	3.26620500	-2.35488000

H	-2.67909600	1.26109500	-1.98596900

C	-0.92871000	4.19286700	-2.22609600

H	1.10210500	4.50187700	-1.50377500

H	-2.92488400	3.57334000	-2.83572800

H	-1.04216200	5.22325900	-2.60195500

I	-2.97913800	-2.36863100	-0.32910900

O	-1.28461100	0.46622900	1.98149000

C	-0.48805500	1.32582300	2.82153300

C	-2.58209300	1.09255600	1.90798600

C	-0.86219200	2.75207100	2.38894600

H	-0.75847900	1.13752300	3.88699800

H	0.57142400	1.05488600	2.65659100

C	-2.30365600	2.60532600	1.82716600

H	-3.10659400	0.66915500	1.02862100

H	-3.15849200	0.82754800	2.82441500

H	-0.17774100	3.09693900	1.58979700

H	-0.79644500	3.46469700	3.23436900

H	-2.34402500	2.94598700	0.77481000

H	-3.05069400	3.17982700	2.40923600

**TS1-Ca**

Coordinates (Angstroms)

X	Y	Z

O	-1.19662700	0.18973100	-1.93270500

O	-0.98280800	-1.90641400	-1.09734400

C	-3.10657900	-0.77155300	-0.77728700

C	-3.68583500	-1.88951600	-0.14333900

C	-3.93474300	0.30872700	-1.14221500

C	-5.05677800	-1.91182100	0.15388500

C	-5.30712500	0.28986200	-0.84603600

C	-5.87357700	-0.81837200	-0.19067500

C	-1.62244200	-0.74917900	-1.09960800

H	-3.02864700	-2.73574300	0.10779300

H	-3.47016500	1.15727200	-1.66631300

H	-5.49685900	-2.78968600	0.65431600

H	-5.94375000	1.14273900	-1.13277200

H	-6.94991000	-0.83450500	0.04318900

Ca	0.76031300	-0.46763000	-0.95200500

C	-1.10196000	0.01940500	0.80347600

C	-1.38966400	1.35399200	1.15047700

C	-0.80665800	-0.87666400	1.84974400

C	-1.35889000	1.79350200	2.48765700

H	-1.65242300	2.07192600	0.35108100

C	-0.75943000	-0.45685500	3.19486400

H	-0.60645000	-1.93667400	1.59953600

C	-1.03692700	0.88455500	3.51452500

H	-1.58436400	2.84487500	2.73707400

H	-0.51056600	-1.17222200	3.99731200

H	-1.00837000	1.22141700	4.56331900

I	3.56128900	-1.45736400	0.04590700

O	1.47722300	1.82825500	-1.10562000

C	0.59657400	2.85986200	-1.61506100

C	2.44830200	2.47977600	-0.26551300

C	0.58667800	3.97905500	-0.54552200

H	1.00250300	3.22914600	-2.58336300

H	-0.38021800	2.36654400	-1.79161500

C	1.62731600	3.51463400	0.50303700

H	2.92955500	1.69635200	0.35310600

H	3.22811900	2.96131500	-0.90204500

H	-0.41496500	4.10400100	-0.09025500

H	0.87082100	4.95036800	-0.99645700

H	1.12050400	3.02386300	1.35834300

H	2.24871400	4.34290000	0.89567600

**IM2-Ca**

Coordinates (Angstroms)

X	Y	Z

O	-0.85937600	-0.41375500	-1.42208100

O	-0.43292300	-0.58759300	0.85547900

C	-2.36268600	-1.73744400	-0.04691100

C	-2.62058300	-2.35808900	1.19158700

C	-2.97044300	-2.26512700	-1.20251000

C	-3.47529400	-3.46982600	1.27692300

C	-3.82680300	-3.37705800	-1.12481800

C	-4.08656600	-3.98249400	0.11779800

C	-1.41512000	-0.49959700	-0.13751500

H	-2.11266600	-1.95850800	2.08294800

H	-2.72860500	-1.78973900	-2.16568700

H	-3.66324700	-3.94660000	2.25314800

H	-4.29091200	-3.78074100	-2.03993600

H	-4.75553700	-4.85557400	0.18209000

Ca	1.16709700	-0.47202400	-0.59041400

C	-2.31999600	0.74847000	0.12681800

C	-3.07517700	1.33368900	-0.90931400

C	-2.38187900	1.32611600	1.40937500

C	-3.86996000	2.46746700	-0.67217800

H	-3.00813100	0.89159900	-1.91516300

C	-3.17482900	2.46097900	1.65498900

H	-1.76424000	0.86832700	2.19740400

C	-3.92382400	3.03740500	0.61376700

H	-4.44784700	2.91709600	-1.49639700

H	-3.20345500	2.90549000	2.66351700

H	-4.54262600	3.92949500	0.80066900

I	4.17343100	-1.05455600	0.21026700

O	1.47595500	1.90632800	-0.79537500

C	0.40382400	2.67432300	-1.40744100

C	2.04829900	2.71871200	0.24697900

C	-0.06368600	3.69201400	-0.34080600

H	0.80064500	3.17940100	-2.31610400

H	-0.37214000	1.93507400	-1.69360400

C	0.83630700	3.39799200	0.88069600

H	2.62143100	2.04883100	0.91920000

H	2.75308900	3.46028300	-0.19884900

H	-1.13532100	3.55927400	-0.09739400

H	0.08086800	4.73007800	-0.70175900

H	0.33188500	2.68695300	1.56599700

H	1.10427300	4.30620900	1.45523700

**IM1-Mg**

Coordinates (Angstroms)

X	Y	Z

O	1.47865100	0.19483900	1.02948800

O	1.50695700	-1.10192600	-0.77382500

C	3.61873700	-0.27940300	0.03497100

C	4.34355900	-0.95839400	-0.96560900

C	4.30595900	0.52911900	0.96337500

C	5.73829700	-0.83061800	-1.03728900

C	5.70071900	0.65854500	0.89040300

C	6.41870500	-0.02126500	-0.10982800

C	2.12229600	-0.41086000	0.10218000

H	3.78730000	-1.58234200	-1.68041600

H	3.72329700	1.05287300	1.73543600

H	6.30009100	-1.36257300	-1.82073200

H	6.23337900	1.29329100	1.61555000

H	7.51368000	0.08094600	-0.16742200

C	-1.00176900	1.37984300	-1.08966100

C	-0.22137800	2.56580000	-1.01073000

C	-2.20027200	1.49843600	-1.84142700

C	-0.60021800	3.77835000	-1.62141900

H	0.72755300	2.54719900	-0.44059400

C	-2.60316000	2.69905000	-2.46054100

H	-2.85964900	0.61606900	-1.95041200

C	-1.80135600	3.84922100	-2.34958800

H	0.03964900	4.67322600	-1.53000300

H	-3.54647100	2.74169900	-3.03226700

H	-2.10912800	4.79307000	-2.82900100

I	-2.02776700	-2.64996100	-0.36728600

O	-1.38634600	0.15276900	1.93155600

C	-0.82114300	1.06809800	2.89509900

C	-2.78436600	0.50924000	1.84040800

C	-1.45578100	2.42459000	2.55846700

H	-1.10455600	0.72532300	3.91698100

H	0.27645600	1.02603700	2.78063700

C	-2.82272000	2.04690900	1.92455400

H	-3.17143300	0.08588900	0.89396300

H	-3.32394900	0.03433300	2.69068500

H	-0.82888800	2.96000300	1.81897400

H	-1.55456400	3.06504000	3.45670100

H	-2.91366500	2.48390800	0.91197400

H	-3.68371700	2.39056100	2.53068500

Mg	-0.37037400	-0.34850300	0.06062400

**TS1-Mg**

Coordinates (Angstroms)

X	Y	Z

O	-0.86415200	-0.46887000	-1.82626500

O	-0.60924600	-1.98809000	-0.17110200

C	-2.83631800	-1.09011800	-0.54459400

C	-3.40763500	-1.87413900	0.47832500

C	-3.68440700	-0.37702600	-1.41538000

C	-4.79960000	-1.92712000	0.64162500

C	-5.07821800	-0.42852500	-1.25382500

C	-5.64121300	-1.20098200	-0.22233200

C	-1.33727300	-1.03735500	-0.72180800

H	-2.72984500	-2.43228400	1.14153600

H	-3.21904700	0.20912100	-2.22155200

H	-5.23616700	-2.54050700	1.44626100

H	-5.73284300	0.13296600	-1.93981800

H	-6.73446000	-1.24199300	-0.09411400

C	-1.01309200	0.54547500	0.71840700

C	-1.55974000	1.82383100	0.48481000

C	-0.58365000	0.25714000	2.03073800

C	-1.64717900	2.79258300	1.50082600

H	-1.93831500	2.07004200	-0.52579700

C	-0.63479900	1.22121300	3.06021000

H	-0.19644700	-0.75676600	2.25588400

C	-1.16960000	2.49388700	2.79296100

H	-2.08288700	3.78562200	1.29416000

H	-0.26882400	0.98122500	4.07338000

H	-1.22747900	3.25004000	3.59257100

I	3.22728200	-1.61940200	0.25096800

O	1.62165000	1.29656800	-1.27188600

C	0.83237100	2.17869400	-2.12084900

C	2.55451200	2.12471500	-0.54009600

C	0.84242500	3.55943100	-1.42610600

H	1.31469000	2.21567600	-3.12137900

H	-0.16154500	1.69946900	-2.21008000

C	1.72952900	3.35484300	-0.17581600

H	2.92917000	1.52842700	0.31359300

H	3.41261000	2.37658400	-1.20465500

H	-0.17806100	3.88236300	-1.14435400

H	1.26771500	4.32904000	-2.10026000

H	1.10017200	3.13080900	0.70917900

H	2.36149600	4.23391600	0.05665500

Mg	0.80762000	-0.52013100	-0.59925700

**IM2-Mg**

Coordinates (Angstroms)

X	Y	Z

O	-0.47308600	-0.39495700	-1.27046300

O	-0.28915200	-0.42834900	1.03393400

C	-2.02724500	-1.77131900	0.00125300

C	-2.35512900	-2.36315300	1.23696200

C	-2.48135000	-2.38635500	-1.18167700

C	-3.12456300	-3.53728400	1.29179400

C	-3.25173500	-3.56081200	-1.13360200

C	-3.57971300	-4.14029700	0.10510600

C	-1.17843700	-0.46936900	-0.05215200

H	-1.96783100	-1.89095800	2.15266000

H	-2.19351900	-1.93002700	-2.14102400

H	-3.36645000	-3.99184800	2.26658800

H	-3.59427800	-4.03304400	-2.06904300

H	-4.18131000	-5.06235500	0.14559000

C	-2.18372000	0.72062900	0.03665000

C	-2.82475500	1.21297100	-1.11802200

C	-2.44967700	1.34668900	1.26967100

C	-3.70288100	2.30695700	-1.04556900

H	-2.59691800	0.73290800	-2.08191300

C	-3.32773400	2.44182200	1.35008000

H	-1.92297400	0.96679500	2.15787100

C	-3.95818900	2.92822100	0.19119900

H	-4.18745300	2.68532700	-1.96053100

H	-3.51596200	2.92574600	2.32251000

H	-4.64238100	3.78960200	0.24967200

I	3.79803900	-1.09837300	0.04815200

O	1.58523000	1.87011600	-0.51126800

C	0.62534400	2.64395300	-1.28600300

C	2.08641400	2.73290800	0.53161700

C	0.04120400	3.69331900	-0.31411100

H	1.16522100	3.11600600	-2.13546100

H	-0.11413200	1.90816500	-1.66098300

C	0.84948000	3.50220700	0.99310400

H	2.55742100	2.09005200	1.30088600

H	2.86391600	3.40763100	0.10508900

H	-1.03901700	3.52211100	-0.14331400

H	0.16675100	4.71544400	-0.72273800

H	0.27536900	2.88469900	1.71231200

H	1.10907800	4.45815500	1.48827600

Mg	1.19671500	-0.18847300	-0.23449400

**10. Characterization of ketone products**

**4-Methylbenzophenone (4a)**

**Yield:** 91%, 35.7 mg, white solid.

**^1^H NMR** (400 MHz, CDCl_3_) δ 7.84 – 7.69 (m, 3H), 7.57 (t, J = 7.4 Hz, 1H), 7.47 (t, J = 7.6 Hz, 2H), 7.28 (d, J = 8.0 Hz, 2H), 2.44 (s, 2H). **^13^C NMR** (101 MHz, CDCl_3_) δ 196.6, 143.4, 138.0, 135.0, 132.3, 130.4, 130.0, 129.1, 128.3, 21.8. **HRMS (ESI)**: [M+H]^+^ Calcd for C_14_H_12_O: 197.0960; found:197.0954. **MIR (cm^-1^):** 2972, 2870, 1648, 1601, 1584, 755.

**3-Methylbenzophenone (4b)**

**Yield:** 85%, 33.3 mg, white solid.

**^1^H NMR** (400 MHz, CDCl_3_) δ 7.81-7.79 (d, J = 8 Hz, 2H), 7.63-7.57 (m, 3H) , 7.50-7.47 (t, J = 6 Hz, 2H), 7.42-7.37 (m, 2H), 2.43 (s, 3H); **^13^C NMR** (101 MHz, CDCl_3_) δ 197.1, 138.3, 137.8, 137.7, 133.3, 132.5, 130.6, 130.2, 128.4, 128.2, 127.5, 21.5. **HRMS (ESI)**: [M+H]^+^ Calcd for C_14_H_12_O: 197.0960; found:197.0941. **MIR (cm^-1^):** 2970, 2870, 1648, 1600, 1580, 1450, 1375, 750.

**2-Methylbenzophenone (4c)**

**Yield:** 78%, 30.6 mg, white solid.

**^1^H NMR** (400 MHz, CDCl_3_) δ 7.80 (d, J = 7.0 Hz, 2H), 7.56 (t, J = 7.4 Hz, 1H), 7.46 (t, J = 7.9 Hz, 2H), 7.43 – 7.34 (m, 1H), 7.29 (t, J = 1.5 Hz, 1H), 7.26 – 7.22 (m, 2H), 2.33 (s, 3H). **^13^C NMR** (101 MHz, CDCl_3_) δ 198.8, 138.7, 137.8, 136.9, 133.3, 131.1, 130.4, 130.3, 128.6, 128.6, 125.3, 20.1. **HRMS (ESI)**: [M+H]^+^ Calcd for C_14_H_12_O:197.0960; found:197.0942. **MIR (cm^-1^):**3060, 2870, 1645, 1600, 1450, 750.

**Benzophenone (4d)**

**Yield:** 82%, 32 mg, white solid.

**^1^H NMR** (400 MHz, CDCl_3_) δ 7.81 (d, *J* = 5.5 Hz, 4H), 7.63 – 7.56 (m, 2H), 7.48 (d, *J* = 7.3 Hz, 4H). **^13^C NMR** (101 MHz, CDCl_3_) δ 196.9, 137.7, 132.5, 130.2, 128.4. **HRMS (ESI)**: [M+H]^+^ Calcd for C_13_H_10_O:183.0804; found:183.0799. Data consistent with literature values[9].

**4-*tert*-Butylbenzophenone (4e)**

**Yield:** 88%, 41.9 mg, colorless oil.

**^1^H NMR** (400 MHz, CDCl_3_) δ 7.85 – 7.73 (m, 3H), 7.58 (t, J = 7.4 Hz, 1H), 7.54 – 7.42 (m, 3H), 1.37 (s, 9H). **^13^C NMR** (101 MHz, CDCl_3_) δ 196.6, 156.3, 138.0, 134.9, 132.3, 130.2, 130.1, 128.3, 125.4, 35.2, 31.2. **HRMS (ESI)**: [M+H]^+^ Calcd for C_17_H_18_O:239.1430; found:239.1428. Data consistent with literature values [10].

**4-Fluorobenzophenone (4f)**

**Yield:** 86%, 34.4 mg, white solid.

**^1^H NMR** (400 MHz, CDCl_3_) δ 7.85 (t, *J* = 7.2 Hz, 2H), 7.77 (d, *J* = 8.3 Hz, 2H), 7.60 (t, *J* = 8.1 Hz, 1H), 7.50 (t, *J* = 8.2 Hz, 2H), 7.23 – 7.13 (m, 2H). **^13^C NMR** (101 MHz, CDCl_3_) δ 195.4, 166.8, 137.6, 133.9, 132.7, 132.6, 130.0, 128.5, 115.7 (d, J = 21.9 Hz). **^19^F NMR** (376 MHz, CDCl_3_) δ-105.83. **HRMS (ESI)**: [M+H]^+^ Calcd for C_13_H_9_OF:201.0710; found:201.0705. Data consistent with literature values [9].

**3-Fluorobenzophenone (4g)**

**Yield:** 70%, 28.1 mg, white solid.

**^1^H NMR** (400 MHz, CDCl_3_) δ 7.79 (d, *J* = 7.1 Hz, 2H), 7.63 – 7.55 (m, 2H), 7.52 – 7.42 (m, 4H), 7.29 (td, 1H). **^13^C NMR** (101 MHz, CDCl_3_) δ 195.5 (d, J = 2 Hz), 161.3 (d, J = 246.5 Hz), 139.7 (d, J = 6.3 Hz), 137.1, 132.9, 130.1, 129.9, 128.5, 125.9 (d, J = 3.0 Hz), 119.4 (d, J = 21.2 Hz), 116.8 (d, J = 22.3 Hz). **^19^F NMR** (376 MHz, CDCl_3_) δ -111.86. **HRMS (ESI)**: [M+H]^+^ Calcd for C_13_H_9_OF:201.0710; found:201.0712. Data consistent with literature values [10].

**4′-Trifluoromethylbenzophenone (4h)**

**Yield:** 85%, 42.5 mg, colorless oil.

**^1^H NMR** (600 MHz, CDCl_3_) δ 7.90 (d, J = 7.8 Hz, 2H), 7.78 (dd, J = 29.7, 7.8 Hz, 4H), 7.63 (t, J = 7.5 Hz, 1H), 7.51 (t, J = 7.7 Hz, 2H). **^13^C NMR** (151 MHz, CDCl_3_) δ 195.6, 140.8, 136.8, 133.1, 130.2, 130.1 (d, J = 4.3 Hz), 128.6, 125.4 (q, J = 3.7 Hz), 122.8 (q, J = 272.6 Hz). **^19^F NMR** (565 MHz, CDCl_3_) δ-63.00. **HRMS (ESI)**: [M+Li]^+^ Calcd for C_14_H_9_OF_3_:257.0760; found: 257.0740. Data consistent with literature values [10].

**Phenyl[4-(trifluoromethoxy)phenyl]methanone (4i)**

**Yield:** 61%, 32.5 mg, colorless oil.

**^1^H NMR** (400 MHz, CDCl_3_) δ 7.87 (d, *J* = 8.5 Hz, 2H), 7.79 (d, *J* = 8.3 Hz, 2H), 7.61 (t, *J* = 6.8 Hz, 1H), 7.50 (t, *J* = 7.7 Hz, 2H), 7.32 (d, *J* = 8.4 Hz, 2H). **^13^C NMR** (101 MHz, CDCl_3_) δ 195.3, 152.2 (q, J = 2.1 Hz), 137.2, 136.0, 132.9, 132.1, 130.1, 128.5, 120.3 (q, J = 259.0 Hz). **^19^F NMR** (376 MHz, CDCl_3_) δ -57.48. **HRMS (ESI)**: [M+H]^+^ Calcd for C_14_H_9_O_2_F_3_:267.0627; found:267.0624. **MIR (cm^-1^):**3060, 2950, 1645, 1580, 1450, 1150, 850.

**Phenyl[3-(trifluoromethoxy)phenyl]methanone (4j)**

**Yield:** 58%, 30.9 mg, colorless oil.

**^1^H NMR** (600 MHz, CDCl_3_) δ 7.80 (d, *J* = 6.8 Hz, 2H), 7.73 (d, *J* = 7.7 Hz, 1H), 7.66 (s, 1H), 7.62 (t, *J* = 7.4 Hz, 1H), 7.56 – 7.47 (m, 3H), 7.45 (d, *J* = 8.3 Hz, 1H). **^13^C NMR** (151 MHz, CDCl_3_) δ 195.0, 149.2 (q, J = 2.1 Hz), 139.5, 136.9, 133.0, 130.0, 129.9, 128.5, 128.4, 124.8, 122.4, 121.3, 120.0 (q, J = 260.0 Hz). **^19^F NMR** (565 MHz, CDCl_3_) δ -57.87. **HRMS (ESI)**: [M+H]^+^ Calcd for C_14_H_9_O_2_F_3_:267.0627; found:267.0615. **MIR (cm^-1^):**3026, 1652, 1275, 1120, 1087, 760.

**4-Chlorobenzophenone (4k)**

**Yield:** 97%, 41.9 mg, colorless oil.

**^1^H NMR** (400 MHz, CDCl_3_) δ 7.83 – 7.72 (m, 4H), 7.60 (t, *J* = 7.4 Hz, 1H), 7.55 – 7.43 (m, 4H). **^13^C NMR** (101 MHz, CDCl_3_) δ 195.6, 139.0, 137.3, 136.0, 132.8, 131.6, 130.0, 128.8, 128.5. **HRMS (ESI)**: [M+H]^+^ Calcd for C_13_H_9_OCl:217.0415; found:217.0414. Data consistent with literature values [10].

**4-methoxybenzophenone (4l)**

**Yield:** 89%, 37.7 mg, colorless oil.

**^1^H NMR** (400 MHz, CDCl_3_) δ 7.83 (d, *J* = 8.8 Hz, 2H), 7.75 (m, 2H), 7.56 (t, *J* = 7.4 Hz, 1H), 7.47 (t, *J* = 7.6 Hz, 2H), 6.96 (d, *J* = 8.9 Hz, 2H), 3.88 (s, 3H). **^13^C NMR** (101 MHz, CDCl_3_) δ 195.7, 163.3, 138.4, 132.7, 132.0, 129.9 (d, *J* = 6.7 Hz), 128.3, 113.7, 55.6. **HRMS (ESI)**: [M+H]^+^ Calcd for C_14_H_12_O_2_:213.0910; found:203.0900. Data consistent with literature values [9].

**(3,5-Dimethoxyphenyl)phenylmethanone (4m)**

**Yield:** 89%, 43.1 mg, colorless oil.

**^1^H NMR** (400 MHz, CDCl_3_) δ 7.82 (d, *J* = 8.6 Hz, 2H), 7.59 (t, *J* = 6.7 Hz, 1H), 7.48 (t, *J* = 7.8 Hz, 2H), 6.93 (s, 2H), 6.68 (d, *J* = 2.7 Hz, 1H), 3.83 (s, 6H). **^13^C NMR** (101 MHz, CDCl_3_) δ 196.5, 160.6, 139.6, 137.6, 132.6, 130.1, 128.4, 108.0, 104.9, 55.5. **HRMS (ESI)**: [M+K]^+^ Calcd for C_15_H_14_O_3_:281.0574; found:281.0570. Data consistent with literature values [11].

**4-Methanethiobenzophenone (4n)**

**Yield:** 95%, 43.3 mg, white solid.

**^1^H NMR** (400 MHz, CDCl_3_) δ 7.81 – 7.72 (m, 4H), 7.61 – 7.55 (m, 1H), 7.48 (t, *J* = 7.5 Hz, 2H), 7.29 (d, *J* = 8.5 Hz, 2H), 2.54 (s, 3H). **^13^C NMR** (101 MHz, CDCl_3_) δ 196.2, 145.6, 138.2, 133.9,132.5,131.0,130.1,128.6,125.1,15.1. **HRMS (ESI)**: [M+H]^+^ Calcd for C_14_H_12_OS:229.0681; found:229.0670. **MIR (cm^-1^):**3058, 2920, 1650, 1188, 660.

**4-(Dimethylamino) benzophenone (4o)**

**Yield:** 78%, 35.1 mg, colorless oil.

**^1^H NMR** (400 MHz, CDCl_3_) δ 7.80 (d, *J* = 9.0 Hz, 2H), 7.72 (d, *J* = 6.9 Hz, 2H), 7.56 – 7.50 (m, 1H), 7.45 (t, *J* = 7.3 Hz, 2H), 6.69 (d, *J* = 9.1 Hz, 2H), 3.07 (s, 6H). **^13^C NMR** (101 MHz, CDCl_3_) δ 195.3, 153.3, 139.4, 132.9, 131.2, 129.6, 128.1, 124.9, 110.7, 40.2. **HRMS (ESI)**: [M+H]^+^ Calcd for C_15_H_15_ON:226.1226; found:226.1217. Data consistent with literature values [12].

**4-Phenylbenzophenone (4p)**

**Yield:** 78%, 40.3 mg, colorless oil.

**^1^H NMR** (400 MHz, CDCl_3_) δ 7.89 (d, J = 8.0 Hz, 2H), 7.84 (d, J = 7.6 Hz , 2H), 7.70 (d, J =8.0 Hz, 2H), 7.65 (d, J = 7.6 Hz, 2H), 7.60 (t, J = 7.4 Hz, 1H), 7.52-7.46 (m, 4H), 7.40(t, J = 7.2 Hz, 1H).**^13^C NMR** (101 MHz, CDCl_3_) δ 196.5, 145.3, 140.0, 137.9, 136.3, 132.5, 130.9, 130.1, 129.1, 128.4, 128.3, 127.4, 127.1. **HRMS (ESI)**: [M+H]^+^ Calcd for C_19_H_14_O:259.1117; found:259.1104. Data consistent with literature values [10].

**2-Benzoylbiphenyl (4q)**

**Yield:** 70%, 36.1 mg, colorless oil.

**^1^H NMR** (400 MHz, CDCl_3_) δ 7.66 (dd, *J* = 8.3, 1.4 Hz, 2H), 7.62 – 7.57 (m, 1H), 7.55 – 7.47 (m, 3H), 7.44 – 7.39 (m, 1H), 7.31 – 7.25 (m, 4H), 7.24 – 7.13 (m, 3H). **^13^C NMR** (101 MHz, CDCl_3_) δ 198.9, 141.2, 140.3, 139.0, 137.5, 132.9, 130.5, 130.2, 130.0, 129.1, 128.9, 128.4, 128.2, 127.4, 127.2. **HRMS (ESI)**: [M+H]^+^ Calcd for C_19_H_4_O:259.1117; found:259.1103. **MIR (cm^-1^):** 3060, 3025, 1650, 1590, 1315, 696.

**1-Benzoylnaphthalene (4r)**

**Yield:** 90%, 41.8 mg, colorless oil.

**^1^H NMR** (400 MHz, CDCl_3_) δ 8.13 – 8.08 (m, 1H), 8.01 (d, *J* = 8.2 Hz, 1H), 7.93 (dd, *J* = 8.1, 1.6 Hz, 1H), 7.88 (dd, *J* = 7.1, 1.4 Hz, 2H), 7.62 – 7.57 (m, 2H), 7.56 – 7.43 (m, 5H). **^13^C NMR** (101 MHz, CDCl_3_) δ 198.2, 138.4, 136.4, 133.8, 133.4, 131.4, 131.1, 130.5, 128.6, 128.5, 127.9, 127.4, 126.6, 125.8, 124.4. **HRMS (ESI)**: [M+H]^+^ Calcd for C_17_H_12_O:233.0961; found:233.0950. Data consistent with literature values [9].

**2-Benzoylnaphthalene (4s)**

**Yield:** 86%, 39.9 mg, colorless oil.

**^1^H NMR** (400 MHz, CDCl_3_) δ 8.27 (d, *J* = 1.1 Hz, 1H), 7.96 (d, *J* = 1.3 Hz, 2H), 7.92 (d, *J* = 8.1 Hz, 2H), 7.87 (dd, *J* = 8.2, 1.4 Hz, 2H), 7.67 – 7.59 (m, 2H), 7.59 – 7.49 (m, 3H). **^13^C NMR** (101 MHz, CDCl_3_) δ 196.9, 138.0, 135.4, 134.9, 132.5, 132.4, 132.0, 130.2, 129.5, 128.5, 128.4, 127.9, 126.9, 125.9. **HRMS (ESI)**: [M+H]^+^ Calcd for C_17_H_12_O:233.0961; found:233.0953. Data consistent with literature values [10].

**9-Phenanthrenylphenylmethanone (4t)**

**Yield:** 75%, 42.3 mg, colorless oil.

**^1^H NMR** (400 MHz, CDCl_3_) δ 8.83 – 8.71 (m, 2H), 8.12 (dd, J = 8.3, 1.3 Hz, 1H), 7.96 (dd, J = 8.3, 1.4 Hz, 2H), 7.93 – 7.83 (m, 2H), 7.79 – 7.57 (m, 5H), 7.48 (t, J = 7.8 Hz, 2H). **^13^C NMR** (101 MHz, CDCl_3_) δ 198.1, 138.3, 135.4, 133.5, 131.4, 130.7, 130.6, 130.2, 129.6, 129.5, 129.3, 128.7, 128.4, 127.3, 127.3, 127.3, 126.7, 123.1, 122.8. **HRMS (ESI)**: [M+H]^+^ Calcd for C_21_H_14_O:283.1117; found:283.1110. **MIR (cm^-1^):**2850, 1816, 1645, 1023, 790, 564.

**2-Benzoylthiophene (4u)**

**Yield:** 86%, 32.3 mg, brown solid.

**^1^H NMR** (400 MHz, CDCl_3_) δ 7.90 – 7.83 (m, 2H), 7.72 (dd, *J* = 5.0, 1.2 Hz, 1H), 7.65 (dd, *J* = 3.8, 1.1 Hz, 1H), 7.59 (t, *J* = 7.4 Hz, 1H), 7.50 (t, *J* = 7.6 Hz, 2H), 7.16 (dd, *J* = 5.0, 3.7 Hz, 1H).**^13^C NMR** (101 MHz, CDCl_3_) δ 188.4, 143.8, 138.2, 135.0, 134.3, 132.4, 129.3, 128.5, 128.1. **HRMS (ESI)**: [M+H]^+^ Calcd for C_11_H_8_OS:189.0369; found:189.0364. Data consistent with literature values [10].

**Benzo[b]thien-2-ylphenylmethanone (4v)**

**Yield:** 90%, 42.9 mg, colorless oil.

**^1^H NMR** (400 MHz, CDCl_3_) δ 7.96 – 7.84 (m, 5H), 7.67 – 7.61 (m, 1H), 7.51 (m, 3H), 7.42 (m, 1H). **^13^C NMR** (101 MHz, CDCl_3_) δ 189.8, 143.2, 142.8, 139.2, 138.0, 132.6, 132.4, 129.4, 128.6, 127.6, 126.2, 125.2, 123.0. **HRMS (ESI)**: [M+H]^+^ Calcd for C_15_H_10_OS:239.0525; found:239.0521. **MIR (cm^-1^):**3058, 1640, 1217, 971, 674.

**2-Benzoylbenzofuran (4w)**

**Yield:** 75%, 33.3 mg, colorless oil.

**^1^H NMR** (400 MHz, CDCl_3_**)** δ 8.05 (d, J = 6.9 Hz, 2H), 7.74 (d, J = 7.9 Hz, 1H), 7.64 (t, J = 7.4 Hz, 2H), 7.59 – 7.48 (m, 4H), 7.34 (t, J = 7.1 Hz, 1H). **^13^C NMR** (101 MHz, CDCl_3_) δ 184.6, 156.1, 152.3, 137.3, 133.0, 129.6, 128.7, 128.5, 127.1, 124.1, 123.4, 116.7, 112.7. **HRMS (ESI)**: [M+H]^+^ Calcd for C_15_H_10_O_2_:223.0754; found:223.0746. Data consistent with literature values [13].

**4-Dibenzofuranylphenylmethanone (4x)**

**Yield:** 62%, 32.6 mg, colorless oil.

**^1^H NMR** (400 MHz, CDCl_3_) δ 8.15 (dd, *J* = 7.7, 1.3 Hz, 1H), 8.00 (d, *J* = 6.5 Hz, 1H), 7.92 (d, *J* = 7.0 Hz, 2H), 7.71 (dd, *J* = 7.6, 1.3 Hz, 1H), 7.63 (t, *J* = 7.4 Hz, 1H), 7.56 – 7.41 (m, 5H), 7.41 – 7.35 (m, 1H). **^13^C NMR** (101 MHz, CDCl_3_) δ 194.1, 156.5, 154.0, 137.8, 133.2, 130.3, 128.8, 128.5, 127.9, 125.7, 124.3, 123.4, 123.3, 122.6, 120.8, 112.3. **HRMS (ESI)**: [M+H]^+^ Calcd for C_19_H_12_O_2_:273.0910; found:273.0899. **MIR (cm^-1^):**3056, 2925, 1660, 1320, 990, 620.

**Dibenzo[b,d]thiophen-4-yl(phenyl)methanone (4y)**

**Yield:** 62%, 35.7 mg, colorless oil.

**^1^H NMR** (400 MHz, CDCl_3_) δ 8.40 (d, *J* = 8.0 Hz, 1H), 8.21 (d, *J* = 9.0 Hz, 1H), 7.96 (dd, *J* = 7.4, 1.8 Hz, 1H), 7.91 (d, *J* = 7.5 Hz, 1H), 7.80 (d, *J* = 7.5 Hz, 2H), 7.58 – 7.48 (m, 6H). **^13^C NMR** (101 MHz, CDCl_3_) δ 196.0, 141.9, 140.9, 138.3, 137.5, 134.1, 132.1, 131.8, 130.4, 129.8, 128.5, 127.4, 125.9, 124.7, 123.8, 123.0, 121.6. **HRMS (ESI)**: [M+H]^+^ Calcd for C_19_H_12_OS:289.0682; found:289.0672. **MIR (cm^-1^):**3060, 2922, 1644, 1320, 1094, 666.

**(1-Methyl-1H-pyrazol-5-yl)phenylmethanone (4z)**

**Yield:** 45%, 16.8 mg, colorless oil.

**^1^H NMR** (400 MHz, CDCl_3_) δ 7.88 (dd, *J* = 8.3, 1.4 Hz, 2H), 7.67 – 7.56 (m, 2H), 7.56 – 7.44 (m, 3H), 4.22 (s, 3H). **^13^C NMR** (101 MHz, CDCl_3_) δ 186.1, 138.2, 137.5, 133.1, 129.6, 128.6, 113.8, 39.9. **HRMS (ESI)**: [M+H]^+^ Calcd for C_11_H_10_ON_2_:187.0866; found:187.0854. **MIR (cm^-1^):**2924, 2202, 1642, 1178, 894, 712.

**Phenyl-3-pyridinylmethanone (4aa)**

**Yield:** 48%, 17.6 mg, colorless oil.

**^1^H NMR** (400 MHz, CDCl_3_) δ 8.98 (dd, *J* = 2.3, 0.9 Hz, 1H), 8.80 (dd, *J* = 4.9, 1.7 Hz, 1H), 8.11 (dt, *J* = 7.8, 2.0 Hz, 1H), 7.80 (dd, *J* = 8.3, 1.4 Hz, 2H), 7.67 – 7.57 (m, 1H), 7.50 (t, *J* = 7.7 Hz, 2H), 7.44 (dd, *J* = 8.0, 4.9 Hz, 1H). **^13^C NMR** (101 MHz, CDCl_3_) δ 195.0, 152.9, 151.0, 137.3, 136.8, 133.3, 130.1, 128.7, 123.5. **HRMS (ESI):** [M+H]^+^ Calcd for C_12_H_9_ON:184.0757; found:184.0753. Data consistent with literature values [14].

**Cyclohexyl phenyl ketone (4ab)**

**Yield:** 60%, 22.6 mg, colorless oil.

**^1^H NMR** (400 MHz, CDCl_3_) δ 7.94 (d, *J* = 7.5 Hz, 2H), 7.68 – 7.40 (m, 3H), 3.26 (tt, *J* = 11.5, 3.3 Hz, 1H), 2.00 – 1.80 (m, 4H), 1.74 (d, *J* = 12.9 Hz, 1H), 1.58 – 1.22 (m, 5H). **^13^C NMR** (101 MHz, CDCl_3_) δ 204.0, 136.4, 132.8, 128.7, 128.4, 45.7, 29.5, 26.1, 26.0. **HRMS (ESI)**: [M+H]^+^ Calcd for C_13_H_16_O:189.1274; found:189.1270. Data consistent with literature values [10].

**1,4-Diphenyl-1-butanone (4ac)**

**Yield:** 49%, 22.0 mg, colorless oil.

**^1^H NMR** (400 MHz, CDCl_3_) δ 7.97 – 7.90 (m, 2H), 7.55 (t, *J* = 7.3 Hz, 1H), 7.45 (t, *J* = 7.6 Hz, 2H), 7.30 (t, *J* = 7.5 Hz, 2H), 7.27 – 7.17 (m, 3H), 2.99 (t, *J* = 7.3 Hz, 2H), 2.73 (t, *J* = 7.6 Hz, 2H), 2.10 (p, *J* = 7.4 Hz, 2H). **^13^C NMR** (101 MHz, CDCl_3_) δ 200.3, 141.8, 137.1, 133.1, 128.6, 128.5, 128.1, 126.1, 37.8, 35.3, 25.8. **HRMS (ESI)**: [M+H]^+^ Calcd for C_16_H_16_O:225.1274; found:225.1278. Data consistent with literature values [15].

**2,2,2-Trifluoro-1-phenylethanone (4ad)**

**Yield:** 25%, 8.7 mg, colorless oil.

**^1^H NMR** (400 MHz, CDCl_3_) δ 8.06 (d, *J* = 1.3 Hz, 2H), 7.71 (t, *J* = 7.5 Hz, 1H), 7.58 – 7.51 (m, 2H). **^13^C NMR** (101 MHz, CDCl_3_) δ 180.8 (q, J = 35.0 Hz), 135.6, 130.2 (q, J = 2.1 Hz), 129.9, 129.2, 118.2 (q, J = 291.3 Hz). **^19^F NMR** (376 MHz, CDCl_3_) δ -71.37. consistent with literature values [16].

**Propiophenone (4ae)**

**Yield:** 30%, 22.0mg, white solid.

**^1^H NMR** (400 MHz, CDCl_3_) δ 7.96 (d, *J* = 7.0 Hz, 2H), 7.55 (t, *J* = 7.4 Hz, 1H), 7.45 (t, *J* = 7.6 Hz, 2H), 3.00 (q, *J* = 7.2 Hz, 2H), 1.23 (t, *J* = 7.2 Hz, 3H). **^13^C NMR** (101 MHz, CDCl_3_) δ 200.8, 137.0, 132.9, 128.6, 128.0, 31.8, 8.3. Data consistent with literature values [17].

**4-Bromobenzophenone (4af)**

**Yield:** 86%, 44.5 mg, colorless oil.

**^1^H NMR** (400 MHz, CDCl_3_) δ 7.76 (d, *J* = 7.0 Hz, 2H), 7.67 (d, *J* = 8.6 Hz, 2H), 7.64 – 7.56 (m, 3H), 7.48 (t, *J* = 7.7 Hz, 2H).**^13^C NMR** (101 MHz, CDCl_3_) δ 195.8, 137.3, 136.4, 132.8, 131.7, 131.7, 130.1, 128.5, 127.6. **HRMS (ESI)**: [M+H]^+^ Calcd for C_13_H_9_OBr:260.9910; found:260.9910. Data consistent with literature values [10].

**4-Iodobenzophenone (4ag)**

**Yield:** 81%, 49.7 mg, colorless oil.

**^1^H NMR** (400 MHz, CDCl_3_) δ 7.85 (d, *J* = 8.4 Hz, 2H), 7.77 (d, *J* = 6.8 Hz, 2H), 7.60 (t, *J* = 7.4 Hz, 1H), 7.55 – 7.45 (m, 4H). **^13^C NMR** (101 MHz, CDCl_3_) δ 196.0, 137.7, 137.2, 137.0, 132.8, 131.6, 130.1, 128.5, 100.2. **HRMS (ESI)**: [M+H]^+^ Calcd for C_13_H_9_OI:308.9770; found:308.9768. Data consistent with literature values [18].

**4,4′-Dimethylbenzophenone (4ah)**

**Yield:** 75%, 31.5 mg, colorless oil.

**^1^H NMR** (400 MHz, CDCl_3_) δ 7.70 (d, J = 7.8 Hz, 4H), 7.27 (d, J = 7.4 Hz, 4H), 2.44 (d, J = 3.6 Hz, 6H).**^13^C NMR** (101 MHz, CDCl_3_) δ 196.4, 143.0, 135.3, 130.3, 129.0, 21.7. **HRMS (ESI)**: [M+H]^+^ Calcd for C_15_H_14_O:211.1117; found: 211.1110. Data consistent with literature values [9].

**(3-Methylphenyl)(4-methylphenyl)methanone (4ai)**

**Yield:** 60%,25.2 mg, colorless oil.

**^1^H NMR** (400 MHz, CDCl_3_) δ 7.72 (d, J = 8.2 Hz, 2H), 7.61 (s, 1H), 7.56 (d, J = 7.2 Hz, 1H), 7.41 – 7.32 (m, 2H), 7.28 (d, J = 7.9 Hz, 2H), 2.43 (d, J = 9.8 Hz, 6H). **^13^C NMR** (101 MHz, CDCl_3_) δ 196.9, 143.3, 138.2, 138.1, 135.1, 133.0, 130.5, 130.4, 129.0, 128.1, 127.3, 21.8, 21.5. **HRMS (ESI)**: [M+H]^+^ Calcd for C_15_H_14_O:211.1117; found: 211.1103. Data consistent with literature values [9].

**2,4′-Dimethylbenzophenone (4aj)**

**Yield:** 45%,18.9 mg, white solid.

**^1^H NMR** (400 MHz, CDCl_3_) δ 7.71 (d, *J* = 8.2 Hz, 2H), 7.41 – 7.35 (m, 1H), 7.32 – 7.23 (m, 5H), 2.43 (s, 3H), 2.32 (s, 3H). **^13^C NMR** (101 MHz, CDCl_3_) δ 198.5, 144.2, 139.1, 136.6, 135.2, 131.0, 130.4, 130.1, 129.3, 128.4, 125.3, 21.8, 20.0. **HRMS (ESI)**: [M+H]^+^ Calcd for C_15_H_14_O:211.1117; found: 211.1120. Data consistent with literature values [9].

**[4-(1,1-Dimethylethyl)phenyl](4-methylphenyl)methanone (4ak)**

**Yield:** 70%, 35.3 mg, colorless oil.

**^1^H NMR** (400 MHz, CDCl_3_) δ 7.77 – 7.71 (m, 4H), 7.49 (d, J = 8.5 Hz, 2H), 7.28 (d, J = 7.9 Hz, 2H), 2.44 (s, 3H), 1.37 (s, 9H). **^13^C NMR** (101 MHz, CDCl_3_) δ 196.4, 156.0, 143.1, 135.3, 135.2, 130.4, 130.1, 129.0, 125.3, 35.2, 31.3, 21.8. **HRMS (ESI)**: [M+H]^+^ Calcd for C_18_H_20_O:253.1587; found: 253.1570. **MIR (cm^-1^):** 2960, 1640, 1294, 1020, 796, 684.

**[4-(Dimethylamino)phenyl](4-methylphenyl)methanone (4al)**

**Yield:** 46%, 22.0 mg, pink solid.

**^1^H NMR** (400 MHz, CDCl_3_) δ 7.79 (d, *J* = 8.9 Hz, 2H), 7.65 (d, *J* = 8.1 Hz, 2H), 7.26 (d, *J* = 7.8 Hz, 2H), 6.68 (d, *J* = 9.0 Hz, 2H), 3.07 (s, 6H), 2.43 (s, 3H). **^13^C NMR** (101 MHz, CDCl_3_) δ 195.1, 153.3, 141.8, 136.6, 132.7, 129.8, 128.8, 125.2, 110.6, 40.2, 21.7. **HRMS (ESI)**: [M+H]^+^ Calcd for C_16_H_17_ON:240.1383; found: 240.1385. **MIR (cm^-1^):**2922, 1658, 1007, 802.

**(4-Methylphenyl)[4-(methylthio)phenyl]methanone (4am)**

**Yield:** 62%, 30.1 mg, colorless oil.

**^1^H NMR** (400 MHz, CDCl_3_) δ 7.71 (dd, J = 17.2, 8.3 Hz, 4H), 7.28 (dd, J = 8.2, 4.7 Hz, 4H), 2.54 (s, 3H), 2.44 (s, 3H). **^13^C NMR** (101 MHz, CDCl_3_) δ 195.7, 145.0, 143.1, 135.2, 134.1, 130.7, 130.2, 129.1, 124.9, 21.7, 15.0. **HRMS (ESI)**: [M+H]^+^ Calcd for C_15_H_14_OS:243.0838; found: 243.0830. **MIR (cm^-1^):**2918, 1645, 1312, 1088, 846, 678.

**4-Methoxy-4′-methylbenzophenone (4an)**

**Yield:** 76%, 34.4 mg, white solid.

**^1^H NMR** (400 MHz, CDCl_3_) δ 7.81 (d, J = 8.9 Hz, 2H), 7.68 (d, J = 8.2 Hz, 2H), 7.27 (d, J = 9.6 Hz, 2H), 6.96 (d, J = 8.9 Hz, 2H), 3.89 (s, 3H), 2.44 (s, 3H). **^13^C NMR** (101 MHz, CDCl_3_) δ 195.4, 163.1, 142.6, 135.5, 132.4, 130.5, 130.0, 128.9, 113.5, 55.5, 21.6. **HRMS (ESI):** [M+H]^+^ Calcd for C_15_H_14_O_2_:227.1067; found:227.1063. Data consistent with literature values [9].

**(3,5-Dimethoxyphenyl)(4-methylphenyl)methanone (4ao)**

**Yield:** 48%, 24.6 mg, colorless oil.

**^1^H NMR** (400 MHz, CDCl_3_) δ 7.73 (d, J = 8.2 Hz, 2H), 7.30 – 7.26 (m, 2H), 6.90 (d, J = 2.4 Hz, 2H), 6.66 (t, J = 2.3 Hz, 1H), 3.82 (s, 6H), 2.44 (s, 3H). **^13^C NMR** (101 MHz, CDCl_3_) δ 196.3, 160.6, 143.4, 139.9, 134.9, 130.4, 129.1, 107.8, 104.7, 55.7, 21.8. **HRMS (ESI):** [M+H]^+^ Calcd for C_16_H_16_O_3_:257.1172; found:257.1170. **MIR (cm^-1^):**2924, 1660, 1188, 1030, 744.

**[1,1′-Biphenyl]-4-yl(4-methylphenyl)methanone (4ap)**

**Yield:** 41%, 22.3 mg, colorless oil.

**^1^H NMR** (400 MHz, CDCl_3_) δ 7.88 (d, *J* = 8.3 Hz, 2H), 7.76 (d, *J* = 8.2 Hz, 2H), 7.70 (d, *J* = 8.3 Hz, 2H), 7.66 (d, *J* = 7.0 Hz, 2H), 7.48 (d, *J* = 7.7 Hz, 2H), 7.41 (t, *J* = 7.3 Hz, 1H), 7.31 (d, *J* = 8.2 Hz, 2H), 2.46 (s, 3H).**^13^C NMR** (101 MHz, CDCl_3_) δ 196.2, 145.1, 143.3, 140.2, 136.7, 135.1, 130.7, 130.4, 129.1, 129.1, 128.2, 127.4, 127.0, 21.8. **HRMS (ESI)**: [M+H]^+^ Calcd for C_20_H_16_O:273.1274; found: 273.1269. Data consistent with literature values [19].

**4-Fluoro-4′-methylbenzophenone(4aq)**

**Yield:** 92%, 39.4 mg, colorless oil.

**^1^H NMR** (400 MHz, CDCl_3_) δ 7.81 (d, J = 4.5 Hz, 2H), 7.68 (d, J = 3.8 Hz, 2H), 7.28 (d, J = 4.6 Hz, 2H), 7.18 – 7.10 (m, 2H), 2.43 (s, 3H). **^13^C NMR** (101 MHz, CDCl_3_) δ 195.2, 166.6, 164.1 (d, J = 253.5 Hz), 143.5, 134.8, 134.2, 132.7 (d, J = 9.1 Hz), 130.3, 129.2, 115.6 (d, J = 21.8 Hz), 21.8. **^19^F NMR** (376 MHz, CDCl_3_) δ -106.27. **HRMS (ESI)**: [M+H]^+^ Calcd for C_14_H_11_OF:215.0867; found: 215.0868. Data consistent with literature values [9].

**(3-Fluorophenyl)(4-methylphenyl)methanone(4ar)**

**Yield:** 42%, 18.0 mg, colorless oil.

**^1^H NMR** (400 MHz, CDCl_3_) δ 7.72 (d, J = 8.3 Hz, 2H), 7.55 (dt, J = 7.6, 1.3 Hz, 1H), 7.53 – 7.40 (m, 2H), 7.33 – 7.24 (m, 3H), 2.45 (s, 3H). **^13^C NMR** (101 MHz, CDCl_3_) δ 195.1, 195.0, 163.7 (d, J = 254 Hz), 161.2, 143.7, 140.1, 140.0, 134.4 (d, J = 8.8 Hz), 130.3, 129.9, 129.9, 129.1, 125.7, 119.3, 119.1, 116.8 (d, J = 21 Hz), 21.7. **^19^F NMR** (376 MHz, CDCl_3_) δ -112.15. **HRMS (ESI)**: [M+H]^+^ Calcd for C_14_H_11_OF:215.0867; found: 215.0870. **MIR (cm^-1^):**2921, 1650, 1118, 850, 628.

**(2-Fluoro-4-methylphenyl)(4-methylphenyl)methanone(4as)**

**Yield:** 53%, 24.2 mg, colorless oil.

**^1^H NMR** (400 MHz, CDCl_3_) δ 7.73 (d, *J* = 6.7 Hz, 2H), 7.44 (t, *J* = 7.6 Hz, 1H), 7.26 (d, *J* = 7.9 Hz, 2H), 7.06 (d, *J* = 6.3 Hz, 1H), 6.96 (d, *J* = 12.4 Hz, 1H), 2.43 (s, 6H). **^13^C NMR** (101 MHz, CDCl_3_) δ 193.3, 160.2 (d, J = 252 Hz), 144.2, 135.3, 130.9, 130.8 (d, J = 10.1 Hz), 130.3, 130.1, 129.2, 125.1 (d, J = 14.2 Hz), 116.9 (d, J = 30 Hz), 21.8, 21.6. **^19^F** **NMR** (376 MHz, CDCl_3_) δ -111.72. **HRMS (ESI)**: [M+H]^+^ Calcd for C_15_H_13_OF:229.1023; found:229.1012. **MIR (cm^-1^):** 3012, 1660, 1220, 1108, 758.

**[3-Fluoro-5-(trifluoromethyl)phenyl](4-methylphenyl)methanone (4at)**

**Yield:** 68%, 38.4 mg, colorless oil.

**^1^H NMR** (400 MHz, CDCl_3_) δ 7.83 (s, 1H), 7.70 (d, *J* = 8.1 Hz, 2H), 7.67 (d, *J* = 9.0 Hz, 1H), 7.54 (d, *J* = 8.2 Hz, 1H), 7.33 (d, *J* = 8.0 Hz, 2H), 2.46 (s, 3H). **^13^C NMR** (101 MHz, CDCl_3_) δ 193.5, 162.2 (d, *J* = 252.5 Hz), 144.5, 141.0, 133.5, 130.2, 129.4, 122.4 (q, *J* = 7.5 Hz), 120.1 (d, *J* = 20.2 Hz), 116.2 (d, *J* = 20.2, 3.8 Hz), 21.7. **^19^F NMR** (376 MHz, CDCl_3_) δ -62.72, -109.23. **HRMS (ESI)**: [M+H]^+^ Calcd for C_15_H_10_OF_4_:283.0740; found: 283.0758. **MIR (cm^-1^):** 1650, 1445, 1210, 987, 542**.**

**(3,5-Difluorophenyl)(4-methylphenyl)methanone (4au)**

**Yield:** 61%, 28.3 mg, colorless oil.

**^1^H NMR** (400 MHz, CDCl_3_) δ 7.71 (d, *J* = 8.1 Hz, 2H), 7.31 (d, *J* = 8.0 Hz, 4H), 7.02 (tt, *J* = 8.5, 2.4 Hz, 1H), 2.45 (s, 3H). **^13^C NMR** (101 MHz, CDCl_3_) δ 193.8, 162.7 (dd, *J* = 262.6, 10.1 Hz), 144.3, 141.0 (t, *J* = 10.1 Hz), 133.8, 130.3, 129.4, 112.9 (dd, *J* = 20.2, 3.0 Hz), 107.5 (d, *J* = 30.3 Hz), 21.8. **^19^F NMR** (376 MHz, CDCl_3_) δ -108.23. **HRMS (ESI)**: [M+H]^+^ Calcd for C_14_H_10_OF_2_:233.0772; found:233.0786. **MIR (cm^-1^):** 3054, 1660, 1120, 878, 750.

**4-(Trifluoromethyl)-4'-methylbenzophenone (4av)**

**Yield:** 68%, 35.9 mg, colorless oil.

**^1^H NMR** (400 MHz, CDCl_3_) δ 7.87 (d, *J* = 8.1 Hz, 2H), 7.73 (dd, *J* = 11.0, 8.2 Hz, 4H), 7.31 (d, *J* = 8.0 Hz, 2H), 2.46 (s, 3H). **^13^C NMR** (101 MHz, CDCl_3_) δ 195.4, 144.2, 141.2, 134.1 (q, J = 32.6 Hz), 130.4, 130.1, 129.3, 125.4 (q, J = 3.7 Hz), 123.7 (q, J = 272.7 Hz), 21.8. **^19^F NMR** (376 MHz, CDCl_3_) δ -62.86. **HRMS (ESI)**: [M+H]^+^ Calcd for C_15_H_11_OF_3_:265.0835;found: 265.0846. Data consistent with literature values [19].

**(4-Methylphenyl)[3-(trifluoromethyl)phenyl]methanone (4aw)**

**Yield:** 68%, 35.9 mg, colorless oil.

**^1^H NMR** (400 MHz, CDCl_3_) δ 8.03 (s, 1H), 7.95 (d, *J* = 7.7 Hz, 1H), 7.83 (d, *J* = 8.1 Hz, 1H), 7.70 (d, *J* = 8.2 Hz, 2H), 7.65 – 7.59 (m, 1H), 7.30 (d, *J* = 7.9 Hz, 2H), 2.45 (s, 3H). **^13^C NMR** (101 MHz, CDCl_3_) δ 195.2, 144.1, 138.7, 134.1, 133.1, 131.1 (q, J = 34.0 Hz), 130.4, 129.4, 129.0, 128.8 (q, J = 3.4 Hz), 126.8 (q, J = 3.8 Hz), 122.5 (q, J = 272.5 Hz), 21.8. **^19^F NMR** (376 MHz, CDCl_3_) δ -62.57. **HRMS (ESI)**: [M+H]^+^ Calcd for C_15_H_11_OF_3_:265.0835; found: 265.0835. **MIR (cm^-1^):** 2924, 1650, 1330, 958, 696.

**(4-methylphenyl)[2-(trifluoromethyl)phenyl]methanone (4ax)**

**Yield:** 42%, 22.2 mg, colorless oil.

**^1^H NMR** (400 MHz, CDCl_3_) δ 7.81 – 7.74 (m, 1H), 7.68 (d, *J* = 8.3 Hz, 2H), 7.65 – 7.58 (m, 2H), 7.41 – 7.34 (m, 1H), 7.26 (d, *J* = 8.1 Hz, 2H), 2.43 (s, 3H). **^13^C NMR** (101 MHz, CDCl_3_) δ 195.2, 144.9, 138.6, 134.0, 131.4, 130.4, 128.8 (q, J = 31.5 Hz), 126.7 (q, J = 272.0 Hz), 21.8. **^19^F NMR** (376 MHz, CDCl_3_) δ -58.08. **HRMS (ESI)**: [M+H]^+^ Calcd for C_15_H_11_OF_3_:265.0835; found: 265.0840. **MIR (cm^-1^):** 2470, 1650, 1020, 964, 792, 636.

**(4-Methylphenyl)[4-(trifluoromethoxy)phenyl]methanone (4ay)**

**Yield:** 75%, 42.1 mg, colorless oil.

**^1^H NMR** (400 MHz, CDCl_3_) δ 7.84 (d, *J* = 8.8 Hz, 2H), 7.71 (d, *J* = 8.2 Hz, 2H), 7.34 – 7.28 (m, 4H), 2.49 (s, 3H). **^13^C NMR** (101 MHz, CDCl_3_) δ 195.1, 152.1, 143.8, 136.3, 134.5, 131.9, 130.3, 129.2, 120.1 (q, *J* = 257 Hz), 21.8. **^19^F NMR** (376 MHz, CDCl_3_) δ -57.48. **HRMS (ESI)**: [M+H]^+^ Calcd for C_15_H_11_O_2_F_3_:281.0784; found: 281.0774. **MIR (cm^-1^):** 2926, 1658, 1254, 1018, 674.

**[3,5-Bis(trifluoromethyl)phenyl](4-methylphenyl)methanone (4az)**

**Yield:** 63%, 41.9 mg, colorless oil.

**^1^H NMR** (400 MHz, CDCl_3_) δ 8.21 (s, 2H), 8.08 (s, 1H), 7.70 (d, *J* = 7.9 Hz, 2H), 7.35 (d, *J* = 7.9 Hz, 2H), 2.48 (s, 3H). **^13^C NMR** (101 MHz, CDCl_3_) δ 193.3, 144.8, 139.8, 133.3, 132.1 (q, *J* = 34.1 Hz), 130.2, 129.8, 129.6, 125.4 (m, *J* = 3.8 Hz), 124.3, 121.6, 21.8. **^19^F NMR** (376 MHz, CDCl_3_) δ -62.78. **HRMS (ESI)**: [M+H]^+^ Calcd for C_16_H_10_OF_6_:333.0709; found:333.0705. **MIR (cm^-1^):** 1668, 1580, 1318, 1198, 698.

**4-Chloro-4′-methylbenzophenone (4ba)**

**Yield:** 65%, 29.9 mg, colorless oil.

**^1^H NMR** (400 MHz, CDCl_3_) δ 7.81 (dd, J = 8.8, 5.4 Hz, 2H), 7.68 (d, J = 8.1 Hz, 2H), 7.28 (d, J = 7.9 Hz, 2H), 7.14 (t, J = 8.7 Hz, 2H), 2.43 (s, 3H). **^13^C NMR** (101 MHz, CDCl_3_) δ 195.2, 166.6, 164.1, 143.5, 134.9, 132.7, 130.3, 129.2, 115.6, 115.4, 21.8. **HRMS (ESI)**: [M+Li]^+^ Calcd for C_14_H_11_OCl:237.0653; found:237.0653. Data consistent with literature values [9].

**(5-Bromo-2-chlorophenyl)(4-methylphenyl)methanone (4bb)**

**Yield:** 60%, 36.9 mg, colorless oil.

**^1^H NMR** (400 MHz, CDCl_3_) δ 7.69 (d, *J* = 8.2 Hz, 2H), 7.53 (dd, *J* = 8.6, 2.4 Hz, 1H), 7.48 (d, *J* = 2.3 Hz, 1H), 7.32 (d, *J* = 8.5 Hz, 1H), 7.27 (d, *J* = 8.0 Hz, 2H), 2.43 (s, 3H). **^13^C NMR** (101 MHz, CDCl_3_) δ 193.3, 145.4, 140.6, 134.0, 133.5, 131.8, 131.6, 130.4, 129.6, 120.6, 21.9. **HRMS (ESI)**: [M+H]^+^ Calcd for C_14_H_10_OBrCl:308.9676; found:308.9685. **MIR (cm^-1^):** 2920, 1667, 1134, 815, 512.

**2,2-Dimethyl-1-(4-methylphenyl)-1-propanone (4bc)**

**Yield:** 42%, 14.8 mg, colorless oil.

**Yield:** 32%, 11.3 mg, colorless oil. (Using pivalic acid as starting material)

**^1^H NMR** (400 MHz, CDCl_3_) δ 7.66 (d, *J* = 8.2 Hz, 2H), 7.20 (d, *J* = 8.0 Hz, 2H), 2.38 (s, 3H), 1.35 (s, 9H). **^13^C NMR** (101 MHz, CDCl_3_) δ 208.5, 141.6, 135.5, 128.8, 128.4, 44.2, 28.3, 21.5. **HRMS (ESI)**: [M+H]^+^ Calcd for C_12_H_16_O:177.1274; found:177.1256. Data consistent with literature values [19].

**Cyclopentyl(4-methylphenyl)methanone (4bd)**

**Yield:** 45%, 17.0 mg, colorless oil.

**Yield:** 24%, 9.1 mg, colorless oil. (Using cyclopentane carboxylic acid as starting material)

**^1^H NMR** (400 MHz, CDCl_3_) δ 7.80 (d, J = 7.9 Hz, 2H), 7.18 (d, J = 7.8 Hz, 2H), 3.62 (p, J = 7.9 Hz, 1H), 2.33 (s, 3H), 1.83 (q, J = 6.8 Hz, 4H), 1.71 – 1.52 (m, 4H). **^13^C NMR** (101 MHz,CDCl_3_) δ 202.6, 143.5, 134.5, 129.3, 128.7, 46.3, 30.1, 26.4, 21.7. **HRMS (ESI)**: [M+H]^+^ Calcd for C_13_H_16_O:189.1274; found:189.1271. Data consistent with literature values [20].

**Cyclohexyl(4-methylphenyl)methanone (4be)**

**Yield:** 62%, 25.1 mg, colorless oil.

**Yield:** 55%, 22.3 mg, colorless oil. (Using cyclohexane carboxylic acid as starting material)

**^1^H NMR** (400 MHz, CDCl_3_) δ 7.84 (d, *J* = 8.2 Hz, 2H), 7.24 (d, *J* = 7.8 Hz, 2H), 3.23 (tt, *J* = 11.5, 3.3 Hz, 1H), 2.39 (s, 3H), 1.85 (d, *J* = 3.3 Hz, 4H), 1.72 (d, *J* = 14.0 Hz, 1H), 1.55 – 1.18 (m, 5H). **^13^C NMR** (101 MHz, CDCl_3_) δ 203.7, 143.6, 133.9, 129.4, 128.5, 45.6, 29.6, 26.1, 26.0, 21.7. **HRMS (ESI)**: [M+H]^+^ Calcd for C_14_H_18_O:203.1430; found:203.1419. Data consistent with literature values [9].

11. **Application expansion**

**Application** **1**: **Synthesis of 4,4′-Difluorobenzophenone**

4,4′-Difluorobenzophenone [21] is not only a drug intermediate for flunarizine, but also a core raw material for PEEK.

**2bf** (0.2 mmol, 1.0 equiv), **3bf** (0.4 mmol, 2 equiv), Ca (0.3mmol, 1.5 equiv) and dry 2-CH_3_THF(0.8 mmol, 4.0 equiv) were added to a stainless-steel milling jar (1.5 mL) with two stainless steel balls (6.0 mm diameter) in glove box and the reaction was carried out in a Retsch MM400 through 30 Hz ball milling. After ball milling for 60 mins, the reaction mixture was quenched with dilute hydrochloric acid. The resulting mixture was then subjected to short-column chromatography on silica gel and eluted with dichloromethane. Removal of the solvent under reduced pressure afforded a residue, which was purified by preparative thin-layer chromatography (PTLC).

**Yield:** 85%, 37.1 mg, white solid.

**^1^H NMR** (400 MHz, CDCl_3_) δ 7.81 (dd, *J* = 8.7, 5.4 Hz, 4H), 7.17 (t, *J* = 8.5 Hz, 4H). **^13^C NMR** (101 MHz, CDCl_3_) δ 193.9, 165.5 (d, *J* = 262.2 Hz), 133.8 (d, *J* = 3.1 Hz), 132.6 (d, *J* = 9.1 Hz), 115.7 (d, *J* = 30.3 Hz). **^19^F NMR** (376 MHz, CDCl_3_) δ -105.63. **HRMS (ESI)**: [M+H]^+^ Calcd for C_13_H_8_OF_2_:219.0616; found: 219.0612. Data consistent with literature values [22].

**Application** **2**: **Synthesis of 5-Bromo-2-chlorophenyl)(4-ethoxyphenyl)methanone**

Dapagliflozin [23-25] is a star drug for the treatment of type II diabetes and has good market prospects. Compound 5-Bromo-2-chlorophenyl)(4-ethoxyphenyl)methanone (**4bg**) is one of the major intermediates in Dapagliflozin synthesis.

**2bg** (0.2 mmol, 1.0 equiv),**3bg** (0.4 mmol, 2 equiv), Ca (0.3mmol, 1.5 equiv) and dry 2-CH_3_THF (0.8 mmol, 4.0 equiv) were added to a stainless-steel milling jar (1.5 mL) with two stainless steel balls (6.0 mm diameter) in glove box and the reaction was carried out in a Retsch MM400 through 30 Hz ball milling. After ball milling for 60 mins, the reaction mixture was quenched with dilute hydrochloric acid. The resulting mixture was then subjected to short-column chromatography on silica gel and eluted with dichloromethane. Removal of the solvent under reduced pressure afforded a residue, which was purified by preparative thin-layer chromatography (PTLC).

**Yield:** 72%, 48.7 mg, white solid.

**^1^H NMR** (400 MHz, CDCl_3_) δ 7.76 (d, *J* = 8.9 Hz, 2H), 7.53 (dd, *J* = 8.5, 2.4 Hz, 1H), 7.48 (d, *J* = 2.4 Hz, 1H), 7.32 (d, *J* = 8.5 Hz, 1H), 6.93 (d, *J* = 8.9 Hz, 2H), 4.11 (q, *J* = 6.9 Hz, 2H), 1.45 (t, *J* = 7.0 Hz, 3H). **^13^C NMR** (101 MHz, CDCl_3_) δ 192.2, 164.0, 140.8, 133. 8, 132.7, 131.6, 131.5, 130.2, 128.7, 120.6, 114.6, 64.0, 14.7. **HRMS (ESI)**: [M+H]^+^ Calcd for C_15_H_12_O_2_BrCl:338.9782; found: 338.9780. **MIR (cm^-1^):**2924, 1658, 1082, 846, 694.

**Application** **3**: **Synthesis of** **1,5-Diphenyl-1-pentanone**

1,5-Diphenyl-1-pentanone [26] is a natural product with good insecticidal activity extracted from Ruixiang wolf poison.

**2bh** (0.2 mmol, 1.0 equiv), **3bh** (0.4 mmol, 2 equiv), Ca (0.3mmol, 1.5 equiv) and dry 2-CH_3_THF (0.8 mmol, 4.0 equiv) were added to a stainless-steel milling jar (1.5 mL) with two stainless steel balls (6.0 mm diameter) in glove box and the reaction was carried out in a Retsch MM400 through 30 Hz ball milling. After ball milling for 60 mins, the reaction mixture was quenched with dilute hydrochloric acid. The resulting mixture was then subjected to short-column chromatography on silica gel and eluted with dichloromethane. Removal of the solvent under reduced pressure afforded a residue, which was purified by preparative thin-layer chromatography (PTLC).

**Yield:** 61%, 29.1mg, white solid.

**^1^H NMR** (400 MHz, Chloroform-*d*) δ 7.95 (d, *J* = 7.5 Hz, 2H), 7.56 (t, *J* = 7.4 Hz, 1H), 7.46 (t, *J* = 7.7 Hz, 2H), 7.31 – 7.26 (m, 2H), 7.20 (d, *J* = 7.1 Hz, 3H), 3.00 (t, *J* = 7.1 Hz, 2H), 2.68 (t, *J* = 7.4 Hz, 2H), 1.86 – 1.78 (m, 2H), 1.76 – 1.67 (m, 2H).**^13^C NMR** (101 MHz, Chloroform-*d*) δ 200.4, 142.4, 137.1, 133.0, 128.7, 128.5, 128.4, 128.1, 125.9, 38.5, 35.9, 31.2, 24.1.**HRMS (ESI)**: [M+Li]^+^ Calcd for C_17_H1_8_O:245.1512; found: 245.1516. Data consistent with literature values [27].

**Application** **4**: **Synthesis of** **[4-(2-Chloroethoxy)phenyl]phenylmethanone**

It is a drug intermediate for synthesizing tamoxifen [28, 29], which is clinically used to treat advanced breast and ovarian cancers.

**2bi** (0.2 mmol, 1.0 equiv), **3bi** (0.4 mmol, 2 equiv), Ca (0.3mmol, 1.5 equiv) and dry 2-CH_3_THF (0.8 mmol, 4.0 equiv) were added to a stainless-steel milling jar (1.5 mL) with two stainless steel balls (6.0 mm diameter) in glove box and the reaction was carried out in a Retsch MM400 through 30 Hz ball milling. After ball milling for 60 min, the reaction mixture was quenched with dilute hydrochloric acid. The resulting mixture was then subjected to short-column chromatography on silica gel and eluted with dichloromethane. Removal of the solvent under reduced pressure afforded a residue, which was purified by preparative thin-layer chromatography (PTLC).

**Yield:** 50%, 26.0 mg, white solid.

**^1^H NMR** (400 MHz, CDCl_3_) δ 7.83 (d, *J* = 8.9 Hz, 2H), 7.76 (d, *J* = 6.8 Hz, 2H), 7.57 (t, *J* = 7.4 Hz, 1H), 7.48 (t, *J* = 7.5 Hz, 2H), 6.98 (d, *J* = 8.8 Hz, 2H), 4.32 (t, *J* = 5.8 Hz, 2H), 3.86 (t, *J* = 5.8 Hz, 2H). **^13^C NMR** (101 MHz, CDCl_3_) δ 195.6, 161.8, 138.2, 132.7, 132.1, 130.9, 129.9, 128.3, 114.2, 68.2, 41.7. **HRMS (ESI)**: [M+H]^+^ Calcd for C_15_H_13_O_2_Cl:261.0677; found:261.0675.

**12. References**

1. I. Priestley, C. Battilocchio, A. V. Iosub, et al. "Safety considerations and proposed workflow for laboratory-scale chemical synthesis by ball milling." *Organic Process Research & Development* 27 (2023): 269–275, <https://doi.org/10.1021/acs.oprd.2c00226>.

2. M. Yamaguchi, H. Shimao, K. Hamasaki, K. Nishiwaki, S. Kashimura and K. Matsumoto. "*gem*-Difluorination of carbon–carbon triple bonds using Brønsted acid/Bu_4_NBF_4_ or electrogenerated acid." *Beilstein Journal of Organic Chemistry* 20 (2024): 2261–2269, <https://doi.org/10.3762/bjoc.20.194>.

3. R. A. Pilli and L. G. Robello. "Palladium-catalyzed double cross-coupling of E-vinylic dibromides with PhZnCl and the synthesis of tamoxifen." *Journal of the Brazilian Chemical Society* 15 (2004): 938–944, <https://doi.org/10.1590/S0103-50532004000600023>

4. M. J. Frisch, G. W. Trucks, H. B. Schlegel, et al. *Gaussian 16, Revision C.01*. Wallingford, CT: Gaussian Inc., 2016. <https://gaussian.com/Citation/>

5. J. M. del Campo, J. L. Gázquez, S. B. Trickey and A. Vela. "Non-empirical improvement of PBE and its hybrid PBE0 for general description of molecular properties." *The Journal of Chemical Physics* 136 (2012): 104108, <https://doi.org/10.1063/1.3691197>.

6. F. Weigend and R. Ahlrichs. "Balanced basis sets of split valence, triple zeta valence and quadruple zeta valence quality for H to Rn: Design and assessment of accuracy." *Physical Chemistry Chemical Physics* 7 (2005): 3297–3305, <https://doi.org/10.1039/B508541A>.

7. F. Weigend. "Accurate Coulomb-fitting basis sets for H to Rn." *Physical Chemistry Chemical Physics* 8 (2006): 1057–1065, <https://doi.org/10.1039/B515623H>.

8. A. V. Marenich, C. J. Cramer and D. G. Truhlar. "Universal solvation model based on solute electron density and on a continuum model of the solvent defined by the bulk dielectric constant and atomic surface tensions." *The Journal of Physical Chemistry B* 113 (2009): 6378–6396, <https://doi.org/10.1021/jp810292n>.

9. M. L. N. Rao, V. Venkatesh and D. Banerjee. "Atom-efficient cross-coupling reactions of triarylbismuths with acyl chlorides under Pd(0) catalysis." *Tetrahedron* 63 (2007): 12917–12926, <https://doi.org/10.1016/j.tet.2007.10.047>.

10. A. T. Biju and F. Glorius. "Intermolecular *N*-heterocyclic carbene catalyzed hydroacylation of arynes." *Angewandte Chemie International Edition* 49 (2010): 9761–9764, <https://doi.org/10.1002/anie.201005490>.

11. J. N. Moorthy and S. Samanta. "Photoinduced C−Br homolysis of 2-bromobenzophenones and pschorr ring closure of 2-aroylaryl radicals to fluorenones." *The Journal of Organic Chemistry* 72 (2007): 9786–9789, <https://doi.org/10.1021/jo7017872>.

12. K. O. Jeon, J. H. Jun, J. S. Yu and C. K. Lee. "Infrared and nuclear magnetic resonance properties of benzoyl derivatives of five-membered monoheterocycles and determination of aromaticity indices." *Journal of Heterocyclic Chemistry* 40 (2003): 763–771, <https://doi.org/10.1002/jhet.5570400504>.

13. G. Kumaraswamy, G. Ramakrishna, R. Raju and M. Padmaja. "An expedient synthesis of enantioenriched substituted (2-benzofuryl)arylcarbinols via tandem Rap–Stoermer and asymmetric transfer hydrogenation reactions." *Tetrahedron* 66 (2010): 9814–9818, <https://doi.org/10.1016/j.tet.2010.10.074>.

14. J. Karthikeyan, K. Parthasarathy and C. H. Cheng. "Synthesis of biarylketones and phthalides from organoboronic acids and aldehydes catalyzed by cobalt complexes." *Chemical Communications* 47 (2011): 10461–10463, <https://doi.org/10.1039/C1CC13771A>.

15. J. A. Murphy, A. G. J. Commeureuc, T. N. Snaddon, et al. "Direct conversion of *N*-methoxy-*N*-methylamides (Weinreb Amides) to ketones via a nonclassical wittig reaction." *Organic Letters* 7 (2005): 1427–1429, <https://doi.org/10.1021/ol050337b>.

16. G. K. S. Prakash, J. B. Hu, M. M. Alauddin, P. S. Conti and G. A. Olah. "A general method of halogenation for synthesis of α-halodifluoromethyl ketones and [18F]-labeled trifluoromethyl ketones." *Journal of Fluorine Chemistry* 121 (2003): 239–243, <https://doi.org/10.1016/S0022-1139(03)00039-3>.

17. A. Shaabani and A. Rahmati. "Aerobic oxidation of alkyl arenes using a combination of *N*-hydroxy phthalimide and recyclable cobalt(II) tetrasulfophthalocyanine supported on silica." *Catalysis Communications* 9 (2008): 1692–1697, <https://doi.org/https://doi.org/10.1016/j.catcom.2007.12.023>.

18. A. Zarei, A. R. Hajipour and L. Khazdooz. "A one-pot method for the iodinationof aryl amines via stable aryl diazonium silica sulfatesunder solvent-free conditions." *Synthesis* 2009 (2009): 941–944.

19. S. Yasuda, H. Yorimitsu and K. Oshima. "Use of aryliron complexes [CpFe(CO)_2_Ar] as arylcarbonyl cation equivalents in the reactions with organolithium reagents to yield ketones." *Organometallics* 28 (2009): 4872–4875, <https://doi.org/10.1021/om900558a>.

20. H. Q. Xiao, X. Z. Shu, K. G. Ji, C. Z. Qi and Y. M. Liang. "An unusual gold-catalyzed rearrangement of α-hydroxy epoxides." *Catalysis Communications* 10 (2009): 1824–1827, <https://doi.org/10.1016/j.catcom.2009.06.008>.

21. G. Krucaite, D. Tavgeniene, M. Kirstukas, et al. "Blue aggregation-induced emission bipolar materials consisting of diphenylsulfone or benzophenone core and triphenylethene-carbazole fragments for highly efficient OLEDs." *Dyes and Pigments* 228 (2024): 112231, <https://doi.org/10.1016/j.dyepig.2024.112231>.

22. Y. J. Su, L. R. Zhang and N. Jiao. "Utilization of natural sunlight and air in the aerobic oxidation of benzyl halides." *Organic Letters* 13 (2011): 2168–2171, <https://doi.org/10.1021/ol2002013>.

23. S. E. Yeoh, K. F. Docherty, P. S. Jhund, et al. "Relationship of dapagliflozin with serum sodium: Findings from the DAPA-HF trial." *Heart Failure* 10 (2022): 306–318, <https://doi.org/10.1016/j.jchf.2022.01.019>.

24. K. P. Garnock Jones. "Saxagliptin/dapagliflozin: A review in Type 2 diabetes mellitus." *Drugs* 77 (2017): 319–330, <https://doi.org/10.1007/s40265-017-0697-1>.

25. S. Dhillon. "Dapagliflozin: A Review in Type 2 Diabetes." *Drugs* 79 (2019): 1135–1146, <https://doi.org/10.1007/s40265-019-01148-3>.

26. H. Zhang, H. Jin, L. Z. Ji, et al. "Design, synthesis, and bioactivities screening of a diaryl ketone-inspired pesticide molecular library as derived from natural products." *Chemical Biology & Drug Design* 78 (2011): 94–100, <https://doi.org/10.1111/j.1747-0285.2011.01082.x>.

27. R. Martínez, D. J. Ramón and M. Yus. "Easy α-alkylation of ketones with alcohols through a hydrogen autotransfer process catalyzed by RuCl_2_(DMSO)_4_." *Tetrahedron* 62 (2006): 8988–9001, <https://doi.org/10.1016/j.tet.2006.07.013>.

28. P. Bhattacharya, B. Abderrahman and V. C. Jordan. "Tamoxifen decreases mortality, but how?" *Journal of Clinical Oncology* 35 (2017): 379, <https://doi.org/10.1200/jco.2016.69.1618>.

29. N. Maani, N. Sabha, K. Rezai, et al. "Tamoxifen therapy in a murine model of myotubular myopathy." *Nature Communications* 9 (2018): 4849, <https://doi.org/10.1038/s41467-018-07057-5>.

**13. NMR Spectra**

**^1^H NMR**-spectrum (400 MHz, CDCl_3_) of **3bh**

**^13^C NMR**-spectrum (101 MHz, CDCl_3_) of **3bh**

**^1^H NMR**-spectrum (400 MHz, CDCl_3_) of **3bi**

**^13^C NMR**-spectrum (101 MHz, CDCl_3_) of **3bi**

**^1^H NMR**-spectrum (400 MHz, Methanol-*d*_4_) of **3cf** and **4cf**

**^1^H NMR**-spectrum (400 MHz, CDCl_3_) of **4a**

**^13^C NMR**-spectrum (101 MHz, CDCl_3_) of **4a**

**^1^H NMR**-spectrum (400 MHz, CDCl_3_) of **4b**

**^13^C NMR**-spectrum (101 MHz, CDCl_3_) of **4b**

**^1^H NMR**-spectrum (400 MHz, CDCl_3_) of **4c**

**^13^C NMR**-spectrum (101 MHz, CDCl_3_) of **4c**

**^1^H NMR**-spectrum (400 MHz, CDCl_3_) of **4d**

**^13^C NMR**-spectrum (101 MHz, CDCl_3_) of **4d**

**^1^H NMR**-spectrum (400 MHz, CDCl_3_) of**4e**

**^13^C NMR**-spectrum (101 MHz, CDCl_3_) of **4e**

**^1^H NMR**-spectrum (400 MHz, CDCl_3_) of **4f**

**^13^C NMR**-spectrum (101 MHz, CDCl_3_) of **4f**

**^19^F NMR**-spectrum (376 MHz, CDCl_3_) of **4f**

**^1^H NMR**-spectrum (400 MHz, CDCl_3_) of **4g**

**^13^C NMR**-spectrum (101 MHz, CDCl_3_) of **4g**

**^19^F NMR**-spectrum (376 MHz, CDCl_3_) of **4g**

**^1^H NMR**-spectrum (400 MHz, CDCl_3_) of **4h**

**^13^C NMR**-spectrum (101 MHz, CDCl_3_) of **4h**

**^19^F NMR**-spectrum (376 MHz, CDCl_3_) of **4h**

**^1^H NMR**-spectrum (400 MHz, CDCl_3_) of **4i**

**^13^C NMR**-spectrum (101 MHz, CDCl_3_) of **4i**

**^19^F NMR**-spectrum (376 MHz, CDCl_3_) of **4i**

**^1^H NMR**-spectrum (400 MHz, CDCl_3_) of **4j**

**^13^C NMR**-spectrum (101 MHz, CDCl_3_) of **4j**

**^19^F NMR**-spectrum (376 MHz, CDCl_3_) of **4j**

**^1^H NMR**-spectrum (400 MHz, CDCl_3_) of **4k**

**^13^C NMR**-spectrum (101 MHz, CDCl_3_) of **4k**

**^1^H NMR**-spectrum (400 MHz, CDCl_3_) of **4l**

**^13^C NMR**-spectrum (101 MHz, CDCl_3_) of **4l**

**^1^H NMR**-spectrum (400 MHz, CDCl_3_) of **4m**

**^13^C NMR**-spectrum (101 MHz, CDCl_3_) of **4m**

**^1^H NMR**-spectrum (400 MHz, CDCl_3_) of **4n**

**^13^C NMR**-spectrum (101 MHz, CDCl_3_) of **4n**

**^1^H NMR**-spectrum (400 MHz, CDCl_3_) of **4o**

**^13^C NMR**-spectrum (101 MHz, CDCl_3_) of **4o**

**^1^H NMR**-spectrum (400 MHz, CDCl_3_) of **4p**

**^13^C NMR**-spectrum (101 MHz, CDCl_3_) of **4p**

**^1^H NMR**-spectrum (400 MHz, CDCl_3_) of **4q**

**^13^C NMR**-spectrum (101 MHz, CDCl_3_) of **4q**

**^1^H NMR**-spectrum (400 MHz, CDCl_3_) of **4r**

**^13^C NMR**-spectrum (101 MHz, CDCl_3_) of **4r**

**^1^H NMR**-spectrum (400 MHz, CDCl_3_) of **4s**

**^13^C NMR**-spectrum (101 MHz, CDCl_3_) of **4s**

**^1^H NMR**-spectrum (400 MHz, CDCl_3_) of **4t**

**^13^C NMR**-spectrum (101 MHz, CDCl_3_) of **4t**

**^1^H NMR**-spectrum (400 MHz, CDCl_3_) of **4u**

**^13^C NMR**-spectrum (101 MHz, CDCl_3_) of **4u**

**^1^H NMR**-spectrum (400 MHz, CDCl_3_) of **4v**

**^13^C NMR**-spectrum (101 MHz, CDCl_3_) of **4v**

**^1^H NMR**-spectrum (400 MHz, CDCl_3_) of **4w**

**^13^C NMR**-spectrum (101 MHz, CDCl_3_) of **4w**

**^1^H NMR**-spectrum (400 MHz, CDCl_3_) of **4x**

**^13^C NMR**-spectrum (101 MHz, CDCl_3_) of **4x**

**^1^H NMR**-spectrum (400 MHz, CDCl_3_) of **4y**

**^13^C NMR**-spectrum (101 MHz, CDCl_3_) of **4y**

**^1^H NMR**-spectrum (400 MHz, CDCl_3_) of **4z**

**^13^C NMR**-spectrum (101 MHz, CDCl_3_) of **4z**

**^1^H NMR**-spectrum (400 MHz, CDCl_3_) of **4aa**

**^13^C NMR**-spectrum (101 MHz, CDCl_3_) of **4aa**

**^1^H NMR**-spectrum (400 MHz, CDCl_3_) of **4ab**

**^13^C NMR**-spectrum (101 MHz, CDCl_3_) of **4ab**

**^1^H NMR**-spectrum (400 MHz, CDCl_3_) of **4ac**

**^13^C NMR**-spectrum (101 MHz, CDCl_3_) of **4ac**

**^1^H NMR**-spectrum (400 MHz, CDCl_3_) of **4ad**

**^13^C NMR**-spectrum (101 MHz, CDCl_3_) of **4ad**

**^19^F NMR**-spectrum (376 MHz, CDCl_3_) of **4ad**

**^1^H NMR**-spectrum (400 MHz, CDCl_3_) of **4ae**

**^13^C NMR**-spectrum (101 MHz, CDCl_3_) of **4ae**

**^1^H NMR**-spectrum (400 MHz, CDCl_3_) of **4af**

**^13^C NMR**-spectrum (101 MHz, CDCl_3_) of **4af**

**^1^H NMR**-spectrum (400 MHz, CDCl_3_) of **4ag**

**^13^C NMR**-spectrum (101 MHz, CDCl_3_) of **4ag**

**^1^H NMR**-spectrum (400 MHz, CDCl_3_) of **4ah**

**^13^C NMR**-spectrum (101 MHz, CDCl_3_) of **4ah**

**^1^H NMR**-spectrum (400 MHz, CDCl_3_) of **4ai**

**^13^C NMR**-spectrum (101 MHz, CDCl_3_) of **4ai**

**^1^H NMR**-spectrum (400 MHz, CDCl_3_) of **4aj**

**^13^C NMR**-spectrum (101 MHz, CDCl_3_) of **4aj**

**^1^H NMR**-spectrum (400 MHz, CDCl_3_) of **4ak**

**^13^C NMR**-spectrum (100 MHz, CDCl_3_) of **4ak**

**^1^H NMR**-spectrum (400 MHz, CDCl_3_) of **4al**

**^13^C NMR**-spectrum (100 MHz, CDCl_3_) of **4al**

**^1^H NMR**-spectrum (400 MHz, CDCl_3_) of **4am**

**^13^C NMR**-spectrum (101 MHz, CDCl_3_) of **4am**

**^1^H NMR**-spectrum (400 MHz, CDCl_3_) of **4an**

**^13^C NMR**-spectrum (101 MHz, CDCl_3_) of **4an**

**^1^H NMR**-spectrum (400 MHz, CDCl_3_) of **4ao**

**^13^C NMR**-spectrum (101 MHz, CDCl_3_) of **4ao**

**^1^H NMR**-spectrum (400 MHz, CDCl_3_) of **4ap**

**^13^C NMR**-spectrum (101 MHz, CDCl_3_) of **4ap**

**^1^H NMR**-spectrum (400 MHz, CDCl_3_) of **4aq**

**^13^C NMR**-spectrum (101 MHz, CDCl_3_) of **4aq**

**^19^F NMR**-spectrum (376 MHz, CDCl_3_) of **4aq**

**^1^H NMR**-spectrum (400 MHz, CDCl_3_) of **4ar**

**^13^C NMR**-spectrum (101 MHz, CDCl_3_) of **4ar**

**^19^F NMR**-spectrum (376 MHz, CDCl_3_) of **4ar**

**^1^H NMR**-spectrum (400 MHz, CDCl_3_) of **4as**

**^13^C NMR**-spectrum (101 MHz, CDCl_3_) of **4as**

**^19^F NMR**-spectrum (376 MHz, CDCl_3_) of **4as**

**^1^H NMR**-spectrum (400 MHz, CDCl_3_) of **4at**

**^13^C NMR**-spectrum (101 MHz, CDCl_3_) of **4at**

**^19^F NMR**-spectrum (376 MHz, CDCl_3_) of **4at**

**^1^H NMR**-spectrum (400 MHz, CDCl_3_) of **4au**

**^13^C NMR**-spectrum (101 MHz, CDCl_3_) of **4au**

**^19^F NMR**-spectrum (376 MHz, CDCl_3_) of **4au**

**^1^H NMR**-spectrum (400 MHz, CDCl_3_) of **4av**

**^13^C NMR**-spectrum (101 MHz, CDCl_3_) of **4av**

**^19^F NMR**-spectrum (376 MHz, CDCl_3_) of **4av**

**^1^H NMR**-spectrum (400 MHz, CDCl_3_) of **4aw**

**^13^C NMR**-spectrum (101 MHz, CDCl_3_) of **4aw**

**^19^F NMR**-spectrum (376 MHz, CDCl_3_) of **4aw**

**^1^H NMR**-spectrum (400 MHz, CDCl_3_) of **4ax**

**^13^C NMR**-spectrum (101 MHz, CDCl_3_) of **4ax**

**^19^F NMR**-spectrum (376 MHz, CDCl_3_) of **4ax**

**^1^H NMR**-spectrum (400 MHz, CDCl_3_) of **4ay**

**^13^C NMR**-spectrum (101 MHz, CDCl_3_) of **4ay**

**^19^F NMR**-spectrum (376 MHz, CDCl_3_) of **4ay**

**^1^H NMR**-spectrum (400 MHz, CDCl_3_) of **4az**

**^13^C NMR**-spectrum (101 MHz, CDCl_3_) of **4az**

**^19^F NMR**-spectrum (376 MHz, CDCl_3_) of **4az**

**^1^H NMR**-spectrum (400 MHz, CDCl_3_) of **4ba**

**^13^C NMR**-spectrum (101 MHz, CDCl_3_) of **4ba**

**^1^H NMR**-spectrum (400 MHz, CDCl_3_) of **4bb**

**^13^C NMR**-spectrum (101 MHz, CDCl_3_) of **4bb**

**^1^H NMR**-spectrum (400 MHz, CDCl_3_) of **4bc**

**^13^C NMR**-spectrum (101 MHz, CDCl_3_) of **4bc**

**^1^H NMR**-spectrum (400 MHz, CDCl_3_) of **4bd**

**^13^C NMR**-spectrum (101 MHz, CDCl_3_) of **4bd**

**^1^H NMR**-spectrum (400 MHz, CDCl_3_) of **4be**

**^13^C NMR**-spectrum (101 MHz, CDCl_3_) of **4be**

**^1^H NMR**-spectrum (400 MHz, CDCl_3_) of **4bf**

**^13^C NMR**-spectrum (101 MHz, CDCl_3_) of **4df**

**^19^F NMR**-spectrum (376 MHz, CDCl_3_) of **4bf**

**^1^H NMR**-spectrum (400 MHz, CDCl_3_) of **4bg**

**^13^C NMR**-spectrum (101 MHz, CDCl_3_) of **4bg**

**^1^H NMR**-spectrum (400 MHz, CDCl_3_) of **4bh**

**^13^C NMR**-spectrum (101 MHz, CDCl_3_) of **4bh**

**^1^H NMR**-spectrum (400 MHz, CDCl_3_) of **4bi**

**^13^C NMR**-spectrum (101 MHz, CDCl_3_) of **4bi**

**14. Substrates incompatible with this reaction**

The reactions were conducted according to General procedure-4, using the iodides shown in Figure S6a with sodium benzoate, or the carboxylate salts shown in Figure S6b with 4-iodotoluene. In addition, the reaction of cyclohexyl iodide with sodium cyclohexanecarboxylate did not afford the desired ketone product (Figure S6c).

Analytical Methods: TLC analysis first revealed no new product spots, with only trace amounts of the starting iodide remaining (approximately 3–5 mg). The reaction mixture was further analyzed by GC-MS or LC-MS, which showed no detectable peak for the target product. Only the characteristic peak of the unreacted starting iodide and peaks corresponding to other complex byproducts were observed.

**Fig. S6. Substrates incompatible with this reaction.**
